# Supplementary material for: An accessory prefrontal cortex–thalamus circuit sculpts maternal behavior in virgin female mice
Source: EMBO J. 2022 Nov 7;41(24):e111648. doi: 10.15252/embj.2022111648 (PMC9753463; doi:10.15252/embj.2022111648)
Supplement: Supplementary file 1 — Expanded View Figures PDF [file EMBJ-41-e111648-s003.pdf]

Expanded View Figures

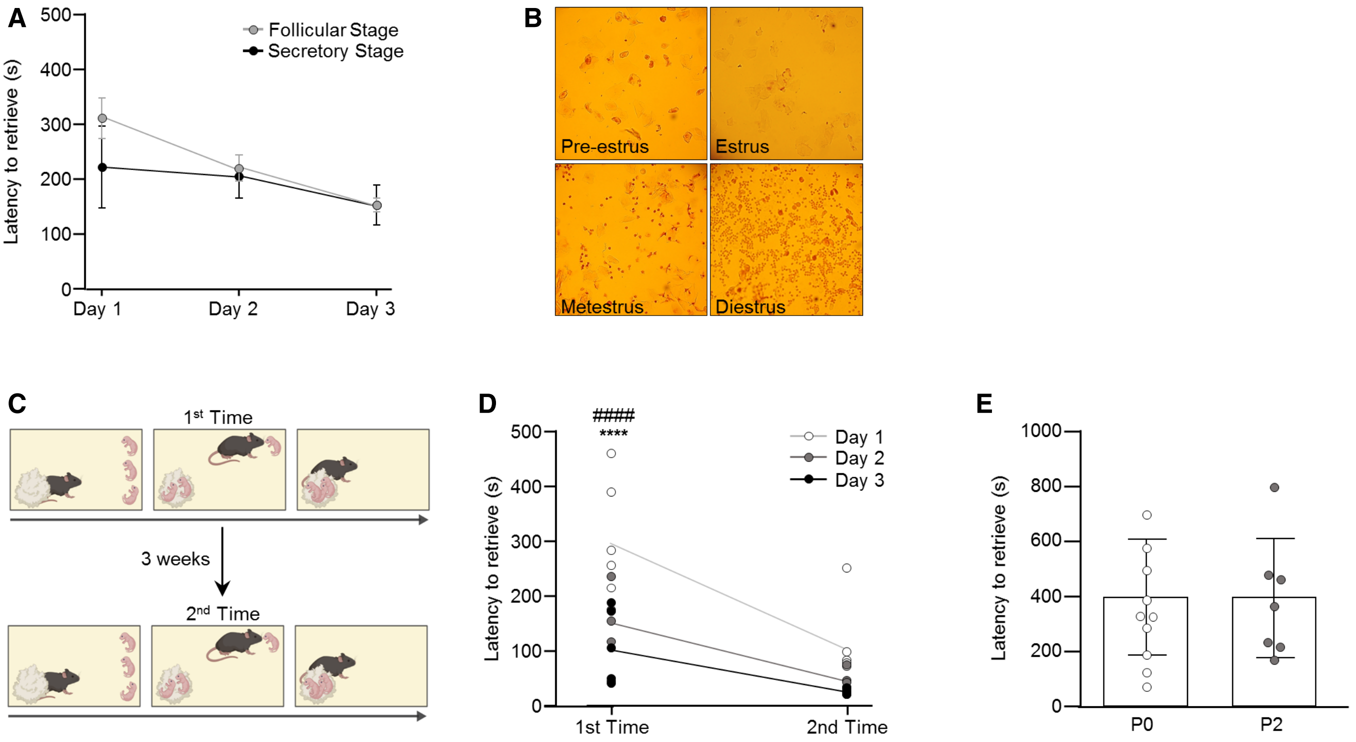

Figure EV1. Pup retrieval behavior in virgin female mice.

A Total pup retrieval time of virgin females in the follicular or secretory stage of the estrus cycle (n = 4/group). Data were analyzed using two-way ANOVA with repeated measures and are expressed as mean ± s.e.m.

B Representative images of vaginal smears of pre-estrus, estrus, metestrus, and diestrus stage.

C Schematic representation of the pup retrieval test at different time points.

D Total pup retrieval time in the first and second rounds of behavioral testing in virgin female mice (n = 6/group). Data were analyzed using two-way ANOVA with repeated measures and are expressed as mean ± s.e.m. ****significantly different from foster mothers, $P < 0.0001$; ####significantly different from mothers, $P < 0.0001$.

E Total pup retrieval time in virgin females exposed to pups at different ages (P0 and P2) on the first day of pup exposure (P0: n = 10: P2: n = 7). Data were analyzed using Student's t-test and displayed as individual values ± s.e.m.

Source data are available online for this figure.

© 2022 The Authors

Figure EV2. ACC projections to different brain structures.

A Schematic illustration of the analyzed thalamic substructures.

B, C Heat-maps of the *z*-scores based on the average ACC output density to the different thalamic (B) and hypothalamic (C) structures.

A

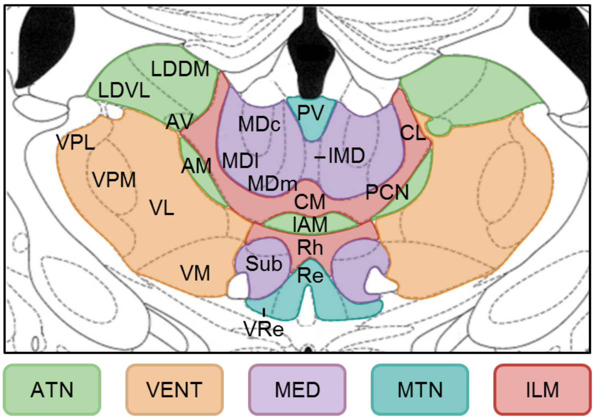

B

Region	Structure	Substructure	Z-score
TH	ATN	AM	2.07
		LD	1.34
		IAD	1.22
		IAM	0.08
		AV	-0.05
		AD	-1.1
	ILM	CL	2.06
		PF	-0.14
		RH	-0.53
		PCN	-1.17
		CM	-1.38
	SPF	SPFp	1.24
		SPFm	0.77
	MED	PR	0.65
		MD	-0.48
		SMT	-1.25
	VENT	VM	0.13
		VAL	-0.09
		SPA	-0.2
		PoT	-0.59
		VPL	-1.29
		VPL	-1.36
	LAT	LP	0.02
		POL	-0.08
	MTN	Xi	-0.13
		RE	-0.43
	GENv	IntG	-0.24
		IGL	-0.98
		SubG	-1.01
		LGv	-1.03
	RT	RT	-0.29
	LH	LH	-0.79

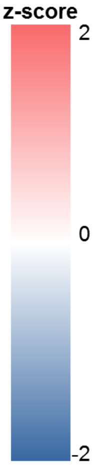

C

Region	Structure	Substructure	Z-score
Hypothalamus	MEZ	PH	0.38
		SUM	0.1
		PMd	-0.87
		PVHd	-0.93
		MM	-1.24
	LZ	ZI	1.91
		STN	-0.31
		PeF	-1.14
		PSTN	-1.18
		LHA	-1.19
		PST	-1.23

© 2022 The Authors

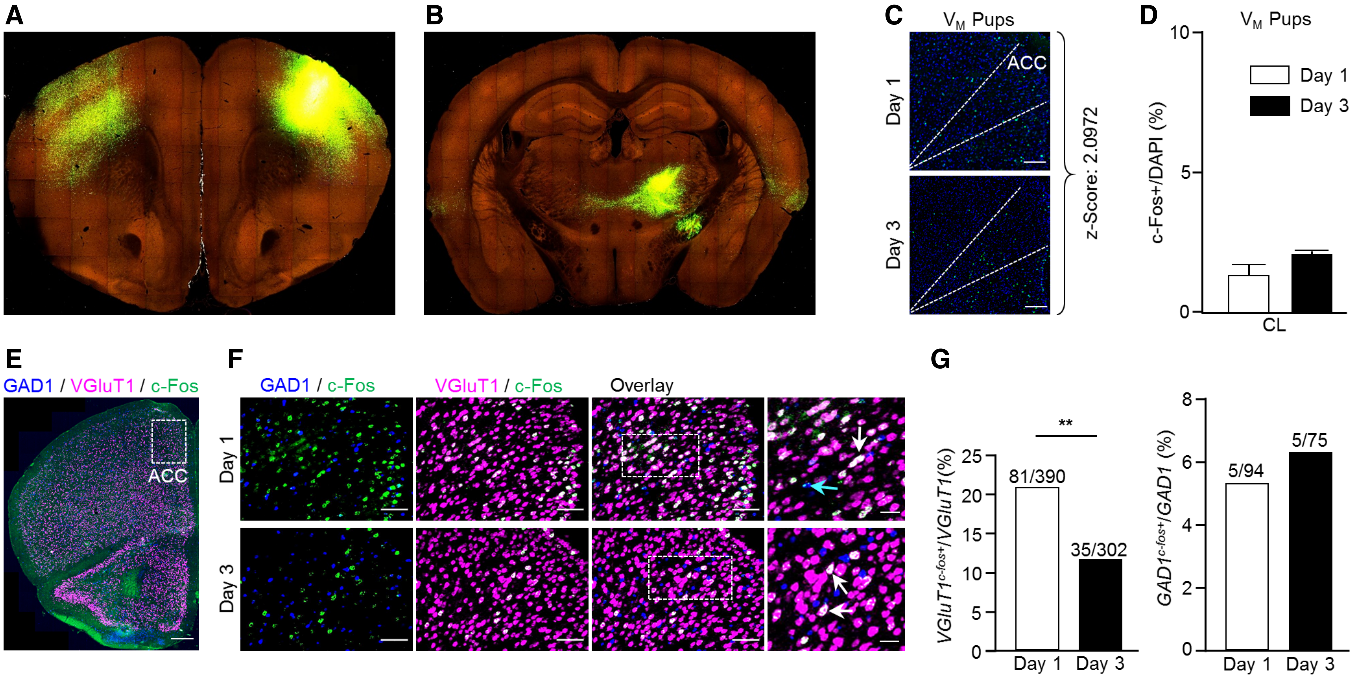

Figure EV3. Projections from the primary motor cortex to CM and PCN.

A, B Representative coronal images from the Allen Mouse Brain Connectivity Atlas (https://connectivity.brain-map.org/projection/experiment/100141780?imageId=102152345), following anterograde labeling with AAV-GFP in the primary motor cortex showing site of injection (A) and axonal distribution pattern in CM and PCN (B).

C Representative coronal sections of the mPFC immunostained for c-Fos with the anatomical borders of the ACC indicated by dashed lines, in virgin male mice on day 1 and 3 of behavioral testing. Scale bar 100 μm, 20× magnification. *Z*-score for the difference in the fraction of c-Fos-expressing cells, between day 1 and 3 of behavioral testing.

D Fraction of c-Fos^+ cells in the CL central lateral nucleus in virgin male mice exposed to pups, on day 1 and 3 of behavioral testing (Day 1: $n = 4$, Day3: $n = 3$). Data were analyzed using Student's *t*-test and are expressed as mean ± s.e.m.

E, F A representative image of the PFC of a virgin female following initial pup exposure showing mRNA expression patterns of *slc17a7* (VGluT1), *gad1* (GAD1), and *c-fos* (c-Fos) together with high-magnification images of the ACC, following the 1st and 3rd days of pup-exposure. The scale bars 500 μm, 10× magnification (A), and 100 μm, 20× magnification (B).

G A comparison of the fraction of excitatory and inhibitory c-Fos^+ cells, of the total VGluT1 and GAD1 cell population in the ACC, following the 1st and 3rd pup exposure days; data were analyzed using Fisher's exact test; $^{**}P < 0.01$.

Source data are available online for this figure.

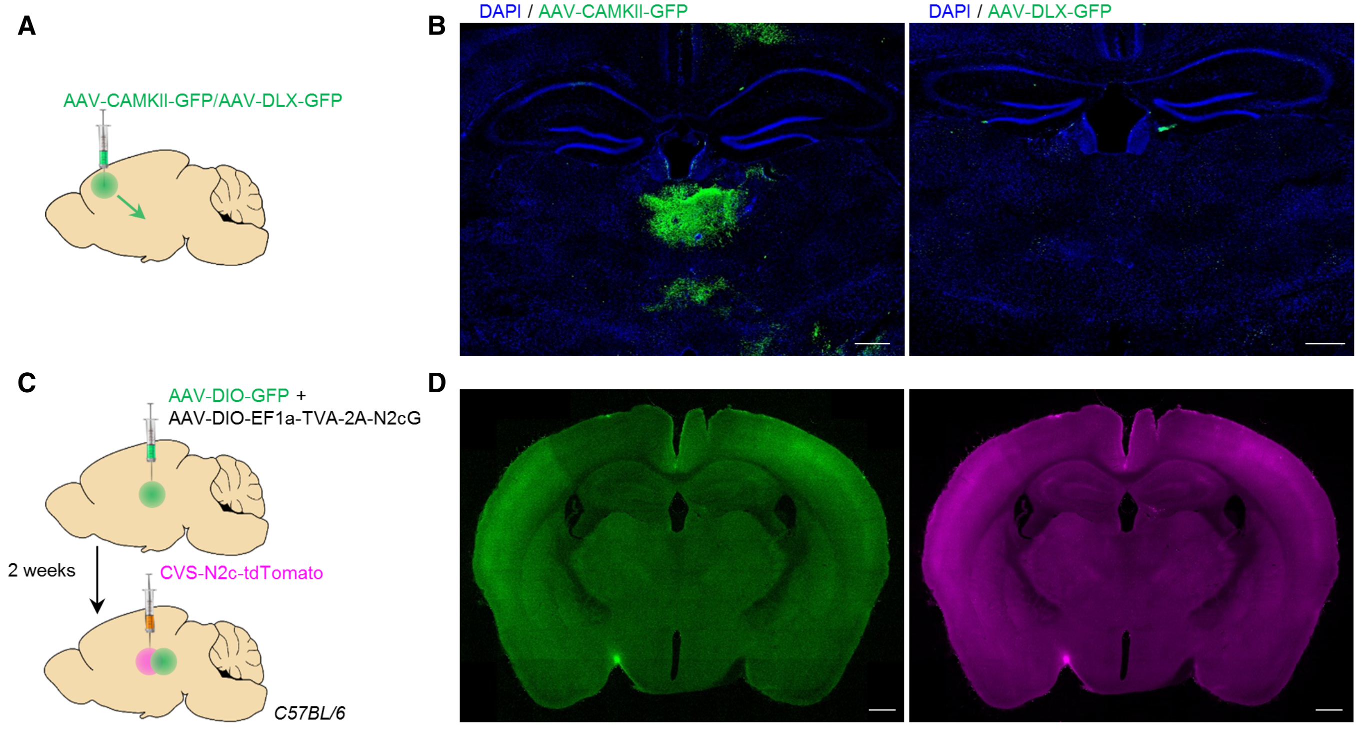

Figure EV4. Target specificity of viral vectors used for anterograde and retrograde labeling.

A Schematic representation of the anterograde tracing from ACC excitatory/inhibitory projections to thalamic structures.

B Representative coronal sections of the thalamic region, following the labeling schemes described in (A), for identification of axonal projections from excitatory (left)—but not inhibitory (right) ACC neurons. Scale bars 200 µm, 10× magnification.

C, D Schematic representation (C) and representative coronal sections (D) of the dual anterograde and retrograde tracing to and from the CL of C57BL/6 control mice. Scale bars 500 µm, 10× magnification.

 © 2022 The Authors

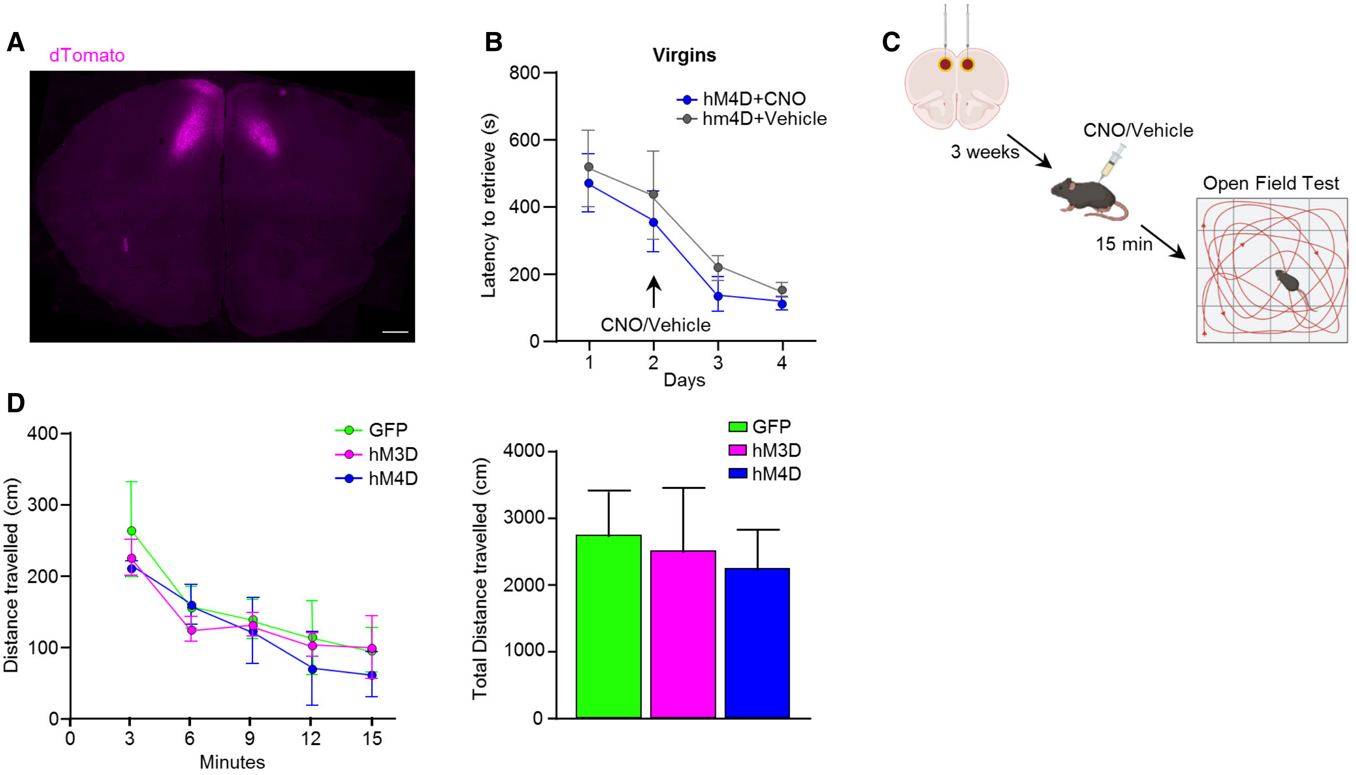

Figure EV5. Chemogenetic strategy.

A Representative coronal section demonstrating AAV-DIO-CAG-hM4D-2A-dTomato expression pattern in the ACC. Scale bar 500 μm, 10× magnification.

B Quantification of pup retrieval latencies of virgin females following CNO/vehicle administration on day 2 in mice injected with hM4D into the ACC. $n = 7$/group. Data were analyzed using two-way ANOVA with repeated measures and are expressed as mean ± s.e.m.

C Schematic representation of the chemogenetic strategy followed by the Open Field Test. Distance traveled in virgin females injected with hM3D/hM4D/GFP after CNO administration.

D Quantification of total distance traveled of virgin females in the Open Field Test following CNO/vehicle administration in mice injected with hM3D or hM4D into the ACC ($n = 5$/group). Data were analyzed using two-way ANOVA with repeated measures and are expressed as mean ± s.e.m.

Source data are available online for this figure.

© 2022 The Authors
