# Supplementary material for: An accessory prefrontal cortex–thalamus circuit sculpts maternal behavior in virgin female mice
Source: EMBO J. 2022 Nov 7;41(24):e111648. doi: 10.15252/embj.2022111648 (PMC9753463; doi:10.15252/embj.2022111648)
Supplement: Supplementary file 2 — Table EV1 [file EMBJ-41-e111648-s006.docx]

**Expanded View Table 1**

**List of names and abbreviations of the brain- structures and**

**substructures receiving input from the ACC.**

| **Structure** | **Region** | **Name** |
| --- | --- | --- |
| Cortical subplate | CLA | Claustrum |
|  | BLA | Basolateral amygdala |
| Hippocampal region | vCA1 | Cornu ammonis subfield 1 (ventral region) |
| Hypothalamus | LZ | Hypothalamic lateral zone |
|  | MEZ | Hypothalamic medial zone |
| Midbrain | MB | Midbrain |
| Pallidum | GPe, GPi | Globus pallidus, external and internal segment |
|  | NDB | Nucleus of the diagonal band of Broca |
| Striatum | CP | Striatum (dorsal region, STRd) |
|  | ACB | Nucleus accumbens (ventral region, STRv) |
| Thalamus | ATN | Anterior group of the dorsal thalamus |
|  | GENv | Geniculate group, ventral thalamus |
|  | ILM | Intralaminar nuclei of the dorsal thalamus |
|  | LAT | Lateral group of the dorsal thalamus |
|  | LH | Lateral habenula (Epithalamus) |
|  | MED | Medial group of the dorsal thalamus |
|  | MTN | Midline group of the dorsal thalamus |
|  | RT | Reticular nucleus of the thalamus |
|  | SPF | Suprafascicular nucleus of the thalamus |
|  | VENT | Ventral group of the dorsal thalamus |
