# Supplementary material for: An accessory prefrontal cortex–thalamus circuit sculpts maternal behavior in virgin female mice
Source: EMBO J. 2022 Nov 7;41(24):e111648. doi: 10.15252/embj.2022111648 (PMC9753463; doi:10.15252/embj.2022111648)
Supplement: Supplementary file 4 — PDF+ [file EMBJ-41-e111648-s005.pdf]

Article

SOURCE DATA | TRANSPARENT PROCESS | OPEN ACCESS

THE EMBO JOURNAL

An accessory prefrontal cortex–thalamus circuit sculpts maternal behavior in virgin female mice

Micaela Glat[1,†], Anna Gundacker[1,†], Laura Cuenca Rico[1], Barbara Czuczu[1], Yoav Ben-Simon[1], Tibor Harkany[2,3,*] & Daniela D Pollak[1,**]

Abstract

The ability to care for the young is innate and readily displayed by postpartum females after delivery to ensure offspring survival. Upon pup exposure, rodent virgin (nulliparous) females also develop parental behavior that over time becomes displayed at levels equivalent to parenting mothers. Although maternal behavior in postpartum females and the associated neurocircuits are well characterized, the neural mechanisms underlying the acquisition of maternal behavior without prior experience remain poorly understood. Here, we show that the development of maternal care behavior in response to first-time pup exposure in virgin females is initiated by the activation of the anterior cingulate cortex (ACC). ACC activity is dependent on feedback excitation by Vglut2$^+$/Galanin$^+$ neurons of the centrolateral nucleus of the thalamus (CL), with their activity sufficient to display parenting behaviors. Accordingly, acute bidirectional chemogenetic manipulation of neuronal activity in the ACC facilitates or impairs the attainment of maternal behavior, exclusively in virgin females. These results reveal an ACC-CL neurocircuit as an accessory loop in virgin females for the initiation of maternal care upon first-time exposure to pups.

Keywords anterior cingulate cortex; centrolateral nucleus of the thalamus; galanin; maternal behavior; virgin females
Subject Category Neuroscience
DOI 10.15252/embj.2022111648 | Received 12 May 2022 | Revised 8 October 2022 | Accepted 14 October 2022 | Published online 7 November 2022
The EMBO Journal (2022) 41: e111648

Introduction

Parental care in altricial mammalian species is critical for the survival of the newborn and constitutes a major determinant of the offspring's physiological, emotional, and cognitive development (Numan & Insel, 2003; Bosch, 2011; Dulac *et al*, 2014; Stockley & Hobson, 2016). Parenting, which comprises a set of species-specific stereotyped behaviors, consists of five major components in rodents: nest building, pup retrieval to the nest, crouching over the young to provide nutrition and warmth, grooming, and defending the pups from any potential threat (Angoa-Pérez & Kuhn, 2015; Kohl *et al*, 2017).

As a form of goal-oriented social behavior, displaying parental care relies on a hierarchical arrangement of neurocircuits, which are synaptically interconnected to allow for the refined and stage-/need-dependent execution of maternal behaviors in response to the dynamics of internal and external states. As such, hormonal and somatic influences together with environmental cues, particularly infant stimuli (e.g., ultrasonic vocalizations) set motivation, arousal, and attraction/drive for the initiation and execution of parental care (Noirot, 1972; D'Amato *et al*, 2005; Brunton & Russell, 2008; McHenry *et al*, 2015).

The surge of estrogen and progesterone during pregnancy primes the onset of maternal behavior immediately at the time of birth in postpartum females (Terkel & Rosenblatt, 1972; Numan & Insel, 2003; Georgescu *et al*, 2021). In the absence of hormonal stimuli, continuous exposure to and interaction with pups (pup sensitization) also leads to the progressive development of parental behaviors in both virgin (nulliparous) female and even male rodents (Rosenblatt, 1967; Stolzenberg & Rissman, 2011). This indicates that the display of maternal care can be inferred as being acquired by experience (Carcea *et al*, 2021) and that hormonal events during pregnancy and parturition merely facilitate the onset of a specific array of actions, but are dispensable for their display *per se*.

The hypothesis that maternal care is, at its core, an innate behavior driven by evolutionarily shaped hard-wired circuits has put maternal behavior in postpartum females in the focus of studies exploring the underlying neural mechanisms (Numan & Insel, 2003; Martyn *et al*, 2012; Tsuneoka, 2019). These studies identified the medial preoptic area (mPOA) of the hypothalamus as a central hub for the coordination of maternal behavior (Numan, 2006; Tsuneoka *et al*, 2013). The documentation of acquired parental behavior in virgin female (Carcea *et al*, 2021) and male (Tachikawa *et al*, 2013) animals nevertheless raises the question of whether the experience-

1 Department of Neurophysiology and Neuropharmacology, Center for Physiology and Pharmacology, Medical University of Vienna, Vienna, Austria
2 Department of Molecular Neurosciences, Center for Brain Research, Medical University of Vienna, Vienna, Austria
3 Department of Neuroscience, Biomedicum 7D, Karolinska Institutet, Solna, Sweden
*Corresponding author. Tel: +46 8 524 87070; E-mail: tibor.harkany@kis.se
**Corresponding author. Tel: +43 1 40160 31270; E-mail: daniela.pollak@meduniwien.ac.at
†These authors contributed equally to this work

© 2022 The Authors. Published under the terms of the CC BY 4.0 license

induced development of parenting could recruit additional non-hypothalamic neuronal circuit modules to support offspring care in the absence of hormonal priming. The existence of such supplementary circuit elements could bear relevance beyond alloparental care to include their recruitment upon insufficient/dysfunctional maternal behavior in postpartum females.

Here, we posited that the acquisition of parental behavior ought to involve the recruitment of prefrontal cortical regions, which regulate the evolvement and maintenance of social and emotional behaviors (Lee *et al*, 2016; Franklin *et al*, 2017). Moreover, we hypothesized that neuronal activity in the prefrontal cortex shall undergo phasic enhancement for virgin females to engage in stereotypic parenting, which could be driven by a hitherto unknown subcortical circuit motif. Therefore, we first used c-Fos-labeling in the prefrontal cortex to single out its anterior cingulate area (ACC) as the domain activated upon the acquisition of care behavior, specifically in virgin females. Viral circuit mapping then reciprocally linked the ACC to the centrolateral nucleus of the thalamus (CL), whose glutamatergic ($Vglut2^+$) neurons are active when virgins engage in maternal care behavior. Molecularly, a galanin $(Gal)^+/Vglut2^+$ neuronal contingent in the CL is innervated by the ACC and selectively activated in virgin females. We then used chemogenetics to show that activation of the ACC-CL circuit facilitates, whereas its inhibition impairs the display of maternal behavior, only in virgin females. Cumulatively, our data suggest causality between neuronal activity within and recurrent feed-forward facilitation between the ACC and CL for the manifestation of parenting behavior in virgin females. We propose ACC-CL connectivity as an accessory to the core hypothalamic circuit of maternal behavior, to launch the display of innate behaviors in naive virgin females, contextually brought about by first-time pup exposure.

Results

Behavioral display of maternal care by virgin females

To determine neuronal circuit substrates of the successful acquisition and expression of maternal behavior, we compared virgin female mice with foster mothers and biological mothers in the pup retrieval test on 3 consecutive days (Fig 1A and B). On day 1, retrieving all pups took significantly longer for virgin females (402 ± 66.2 s) as compared to biological mothers (66.5 ± 18.8 s) and foster mothers (56.8 ± 20.8 s). This difference became significantly reduced on the 2nd (167.9 ± 61.1 s) and 3rd days (72.8 ± 9.2 s; virgins vs. mothers and foster mothers $P = 2.9 \times 10^{-7}$ and $P = 1.9 \times 10^{-8}$, respectively (day 1) and $P = 0.1$ (day 3); Fig 1B). No difference between mothers and foster mothers was found on any of the testing days. Foster mothers were therefore used in all ensuing experiments in comparison to virgin females, particularly to exclude pup-driven bias.

To further characterize the development of parental responsiveness in virgin females, we monitored the dynamic appearance of pup-directed behaviors over 3 consecutive days and observed an increase in the percentage of time spent in pup-directed behavior (including crouching, licking and grooming, and covering over the pups) during 15-min epochs (343.4 ± 49.9 s (day 1); 633.7 ± 53.3 s (day 3); $n = 7$/group; day 1 vs. day 3: $P = 0.017$; Fig 1C). In parallel, a decrease in non-pup-directed behavior was seen across the days (39.7 ± 13.4 s (day 1); 5.3 ± 3.7 s (day 3); $n = 7$/group; day 1 vs. day 3: $P = 0.011$; Fig 1D). These data suggest that virgins can rapidly acquire maternal care behavior even in the absence of long-lived hormonal influences associated with pregnancy. To determine whether variations in hormonal changes associated with the different stages of the ovarian cycle affected the acquisition of parental behavior in virgin females, we grouped mice into those in "follicular stage" (pre-estrus/estrus) and those in "secretory stage" (metestrus/diestrus) on the first day of pup exposure. A statistical difference between groups was not observed, suggesting that hormonal fluctuations do not bias the performance of virgin females (Fig EV1A and B).

Next, we tested whether virgin females were able to retain the learned parenting behavior over time and conducted a second pup retrieval test 3 weeks after the first one (Fig EV1C). In the second test, virgin females were fast in retrieving the pups (97.8 ± 26.8 s (second round, day 1); $n = 7$/group; $P = 7.7 \times 10^{-7}$; Fig EV1D). These data suggest that the acquired display of maternal care behavior is irrevocable in pup-experienced females.

Pup-to-mother communication does not bias the behavioral display of virgin females

Parental responsiveness is determined by the bidirectional communication between offspring and parent (Swain *et al*, 2004). In the pup retrieval paradigm, ultrasonic vocalization emitted by the pups evokes and reinforces care behavior (Wöhr *et al*, 2010; Wöhr & Schwarting, 2010; Portfors & Perkel, 2014). Considering that the complexity of the patterns of pup vocalizations is developmentally regulated (Grimsley *et al*, 2011), we tested if the age of the pups, and henceforth their calls, could affect parenting by virgin females over time. Exposure to pups P0 vs. P2 of age did not impact the performance of virgin females on the 1st day of testing (P0: 402 ± 66 s and P2: 397.14 ± 82 s; $n = 10$ (P0) and $n = 7$ (P2); $P = 0.96$; Fig EV1E). Thus, we posited that the progressive development of maternal behavior could be driven by physiological changes in the virgin females' brains rather than by changes in pup-emitted stimuli.

Neuronal activation upon acquired maternal behavior in virgin females

The neural network underpinning the ability to acquire and display parental responsiveness in virgin females is unknown. Here, we mapped the expression of the immediate early-gene c-Fos, a surrogate of neuronal activation, upon pup retrieval after the 1st and last days of testing to screen for the temporal association between neuronal activation and the dynamic changes in behavioral outcomes. We compared virgin females (V_{pups}) to foster mothers (FM_{pups}) and included virgin females exposed to objects (V_{obj}) as a control group ($n = 5/FM_{pups}$, $n = 6/V_{pups}$, $n = 7/V_{obj}$) to filter out novelty-induced rather than goal-directed neural activation (Fig 1E and F). The density of c-Fos-labeled cells was calculated as the fraction of all DAPI$^+$ cells (% c-Fos$^+$ cells; see Materials and Methods) in 12 brain regions purportedly involved in controlling parental behavior (Dulac *et al*, 2014). For each of

 © 2022 The Authors

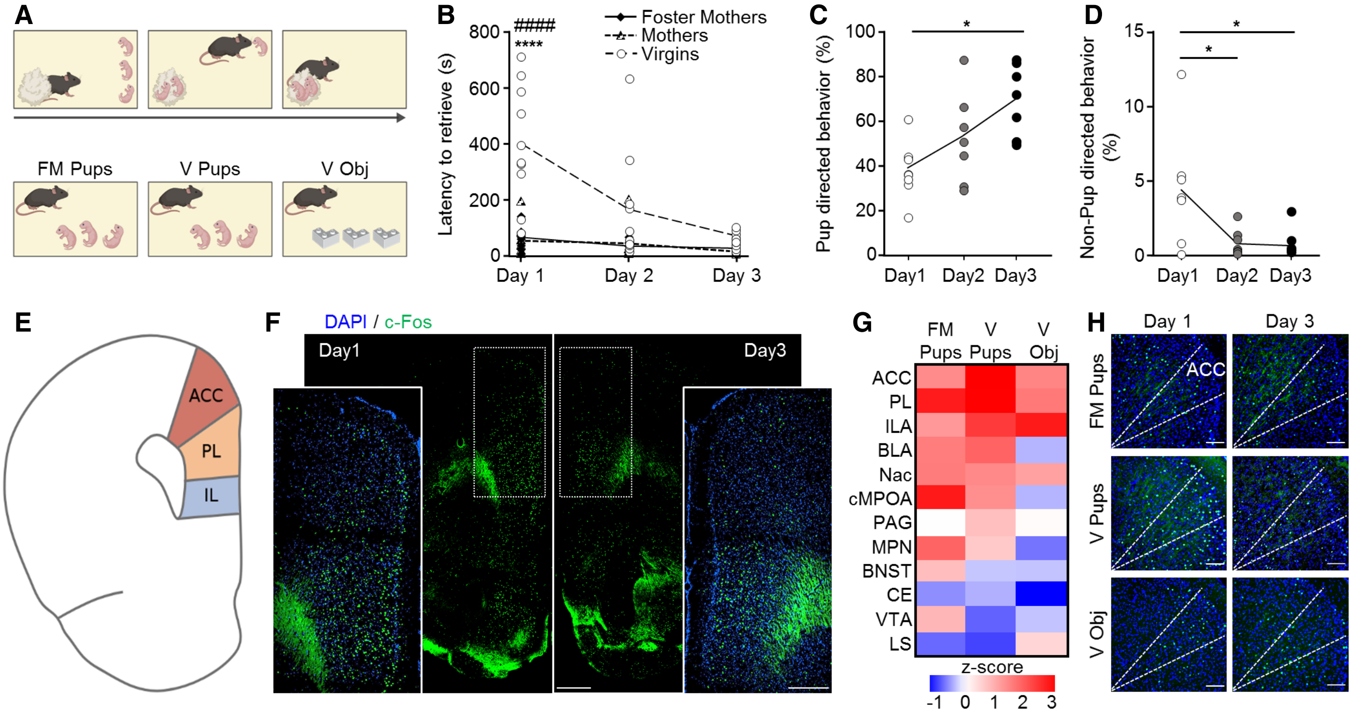

Figure 1. Dynamics of experience-dependent parental behavior in virgin female mice and corresponding neuronal activity patterns.

A Schematic representation of the pup retrieval test.

B Total pup retrieval time in biological mothers ($n = 6$), foster mothers ($n = 7$), and virgin females ($n = 10$) on 3 consecutive days. Data were analyzed using mixed-model ANOVA with repeated measures and are displayed as mean ± s.e.m.; ****significantly different from foster mothers, $P < 0.0001$; ####significantly different from mothers, $P < 0.0001$.

C, D Fraction of pup-directed (C) and non-pup-directed (D) behaviors of virgin females of the total session duration in each of the 3 pup exposure days ($n = 7$). Data were analyzed using two-way ANOVA with repeated measures and are displayed as individual measurements with the line connecting the mean value; *$P < 0.05$.

E Schematic representation of a coronal section showing regions of the medial prefrontal cortex (mPFC).

F Representative image of a coronal brain section immunostained for c-Fos after pup retrieval on day 1 and 3. The mPFC region is indicated by dashed lines, which are also shown in the insets in higher magnification. The scale bar indicates 500 μm, 10× magnification.

G Heat-map of the *Z*-score for the difference in the fraction of c-Fos-expressing cells, between day 1 and 3 of behavioral testing. Foster mothers (FM Pups; $n = 5$), virgins with pups (V Pups; $n = 6$), and virgins with object (V Obj; $n = 7$).

H Representative coronal sections of the mPFC immunostained for c-Fos with the anatomical borders of the ACC indicated by dashed lines, in the 3 groups described in (G) on day 1 and 3 of behavioral testing. Scale bar 100 μm, 20× magnification.

Source data are available online for this figure.

the 3 groups, *z*-scores were calculated (Δ day 3−day 1 of c-Fos$^+$ cells, expressed as a percentage) in order to demonstrate changes across time (Fig 1G). The medial prefrontal cortex (mPFC), subdivided into the anterior cingulate (ACC), prelimbic (PL), and infralimbic (ILA) cortices exhibited the greatest change in neural activity in virgin females exposed to pups, including a gradual decrease in neural activity over the days of testing. Specifically, the *z*-score of the ACC distinguished V_{pups} from the other groups (*z*-scores: $V_{pups} = 3.89$, $FM_{pup} = 1.75$, $V_{obj} = 1.85$; Fig 1H). In contrast, more subtle and non-specific changes were observed in the basolateral amygdala (BLA), central amygdala (CE), nucleus accumbens (Nac), the central part of the medial preoptic area (cMPOA), medial preoptic nucleus (MPN), bed nucleus of stria terminalis (BNST), periaqueductal gray (PAG), ventral tegmental area (VTA), and lateral septum (LS). These data suggest a selective and specific activation of mPFC subregions during the acquisition of maternal behavior in virgin females.

The acquisition of parental care in virgin females recruits an ACC–thalamic circuit

To identify the extent of the neuronal network with the ACC being its cortical hub in virgin females, we first mapped the input/output connectivity of ACC neurons. We relied on open-source data from the Allen Mouse Brain Connectivity Atlas (https://connectivity.brain-map.org/static/brainexplorer) to map ACC efferents (Oh *et al*, 2014). We chose 3 series of experiments, in which the ACC was exclusively targeted for anterograde labeling (with minimal dispersion of tracers in neighboring cortical areas). These data revealed brain-wide ACC innervation patterns, with thalamic nuclei serving as major postsynaptic targets (Fig 2A).

Next, we resolved the origins of inputs to the ACC by using G-deleted, envA-pseudotyped CVS-N2c rabies viral vectors ($RVdG_{envA}$-CVS-N2c) amenable to transsynaptic retrograde labeling (Fig 2B and C). Besides unidirectional monosynaptic inputs from the claustrum

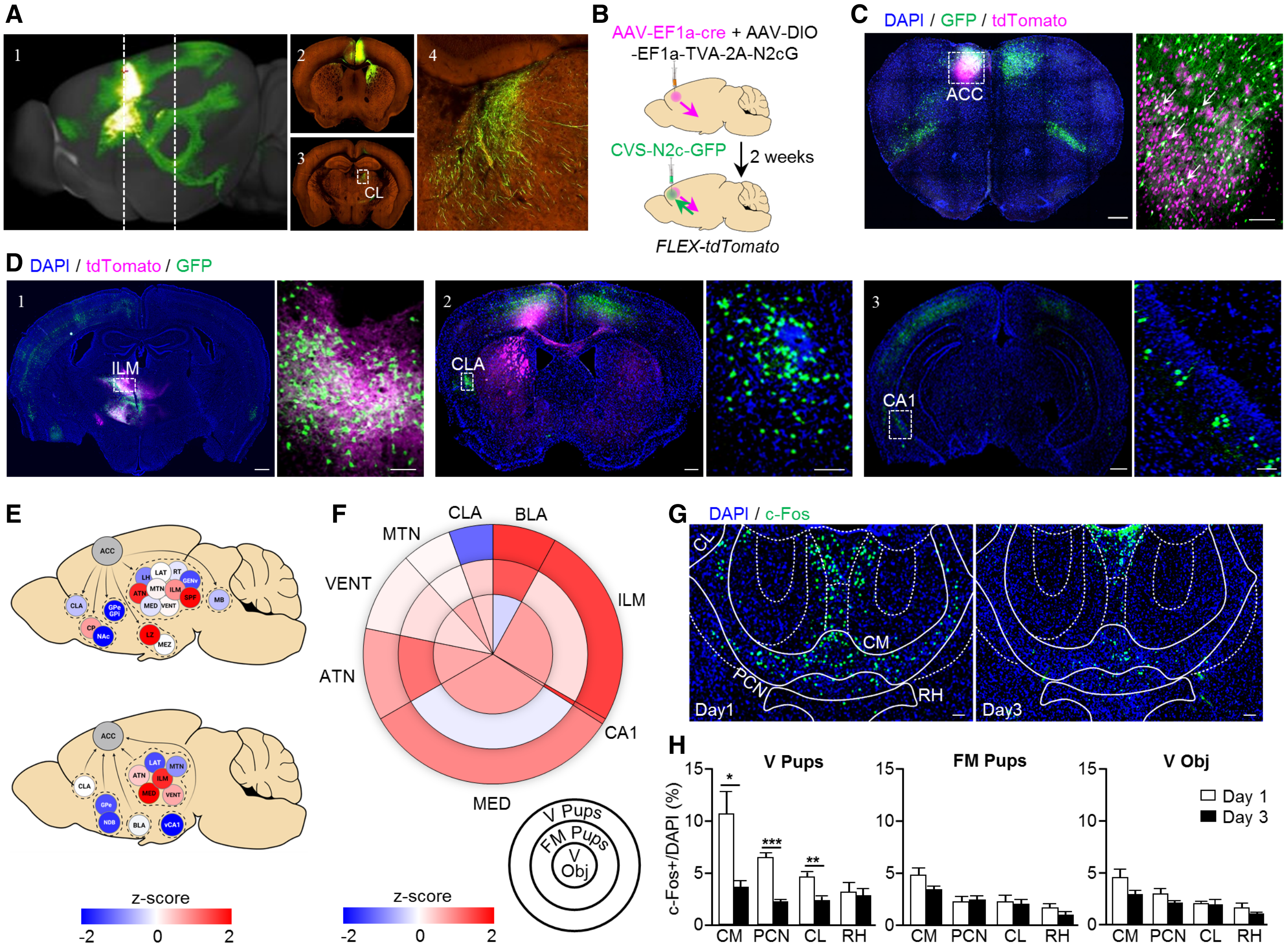

Figure 2. Identification of prefrontal–thalamic circuit changes during experience-induced parental behavior in virgin females.

A Reconstruction of the mouse brain following anterograde labeling with AAV-GFP in the ACC (1); representative coronal images showing the site of injection and axonal distribution pattern in the dorsal striatum (2) and central lateral nucleus of the thalamus (3, 4). Images in A1–A4 were taken from the Allen Mouse Brain Connectivity Atlas, https://connectivity.brain-map.org/projection/experiment/139520203.

B Schematic representation of the dual anterograde and retrograde tracing to and from the ACC, in FLEX/tdTomato mice.

C Representative coronal section showing a labeling pattern at the site of injection. White arrows in the magnified image (C, right) point to double-labeled putative starter cells for transsynaptic retrograde labeling. The scale bar indicates 500 μm, 10× magnification (left image), and 100 μm, 20× magnification (right image).

D Representative coronal sections showing bidirectional connectivity patterns with the thalamus (1) and monosynaptic input to the ACC from the claustrum (2) and ventral CA1 (3). Scale bars 500 μm, 10× magnification (left images), and 100 μm, 20× magnification (right images).

E Graphic representation and *z*-scores of ACC synaptic outputs (top, $n = 3$) and inputs (bottom, $n = 4$). ACC anterior cingulate cortex, ATN anterior group of the dorsal thalamus, BLA basolateral amygdala, CLA claustrum, CP caudo-putamen, GENv geniculate group-ventral part, GPe globus pallidus-external segment, GPi globus pallidus-internal segment, ILM intralaminar nucleus of the thalamus, LAT lateral group of the dorsal thalamus, LH lateral habenula, LZ hypothalamic lateral zone, MB midbrain, MED medial group of the dorsal thalamus, MEZ hypothalamic medial zone, MTN midline group of the dorsal thalamus, NAc nucleus accumbens, RT reticular nucleus of the thalamus, SPF subparafascicular nucleus, and VENT ventral group of the dorsal thalamus.

F Infographic showing the relative proportion of projection density to ACC for each region in a form of a pie chart and *z*-scores for the difference in c-Fos expression between the first and the last day of behavioral testing in brain regions projecting to the ACC represented in a form of heat-map.

G Representative coronal sections of the dorsal thalamus, following c-Fos immunolabeling on day 1 (left) and day 3 (right), with the ILM subnuclei indicated; CM central medial nucleus, CL central lateral nucleus, PCN paracentral nucleus, RH rhomboid nucleus. Scale bar 200 μm, 10× magnification. Foster mothers ($n = 5$), virgins with pups ($n = 6$), and virgins with object ($n = 7$).

H Fraction of c-Fos^{+} cells in different ILM subnuclei, virgin exposed to pups (left), in foster mothers (middle), and virgin exposed to object (right), on day 1 and 3 of behavioral testing. Foster mothers ($n = 5$), virgins with pups ($n = 6$), and virgins with object ($n = 7$). Data were analyzed using two-way ANOVA with repeated measures and are expressed as mean ± s.e.m.; $*P < 0.05$, $**P < 0.01$, $***P < 0.001$.

Data information: Images and data shown in (A, E) were taken from the Allen Mouse Brain Connectivity Atlas.
Source data are available online for this figure.

 © 2022 The Authors

and ventral hippocampal CA1 region, we demonstrate that the ACC is reciprocally connected with the thalamus (Fig 2D). The relative contributions of these brain regions were scaled by *z*-scores that quantified the signal density for each tested region, with a focus on ACC, cortical plate, and subcortical structures (Figs 2E and EV2A–C) in both anterograde and retrograde labeling experiments. We find that among the directionally connected thalamic regions, the intralaminar nuclei of the dorsal thalamus (ILM) provides a major excitatory input to the ACC (Kung & Shyu, 2002; Barroso-Chinea *et al*, 2008; Fig 2E and F and for neuronal phenotypes see Fig 3).

We then asked if any of the brain regions projecting to the ACC exhibits an activation pattern reminiscent of the c-Fos labeling we have seen in the ACC during pup retrieval. Therefore, we determined changes in the relative density of c-Fos$^+$ cells across days (Δ day 3−day 1), selectively in virgin females exposed to pups, and then ranked the identified regions according to their contribution to the total of ACC projections (Fig 2F). This analysis revealed that the ILM is not only providing the densest projection to the ACC but also is specifically activated in virgin females displaying maternal care behavior. A further anatomical subdivision of the ILM showed a significant decrease in c-Fos labeling across days in its centromedial (CM; P = 0.04), paracentral (PCN; P = 0.0007), and centrolateral (CL; P = 0.004) subnuclei ($n = 5/FM_{\text{pups}}$, $n = 6/V_{\text{pups}}$, $n = 7/V_{\text{obj}}$; Fig 2G and H). A focused analysis of the connectivity patterns showed that both the CM and PCN primarily receive afferents from the motor cortex (Fig EV3). In contrast, the CL is innervated by the ACC (Fig EV2B). Based on our activity profiling and connectome data we identify the CL as the only area specifically and bidirectionally connected to the ACC, and participating in the acquisition of maternal behavior in virgin females.

We next tested if the same neurocircuit was also recruited in virgin male animals upon repeated pup exposure. To this end, we evaluated the relative density of ACC c-Fos$^+$ neurons across days of pup exposure (Δ of day 3−day 1) as before in female mice. We found that the *z*-score of the ACC in virgin males (2.09) was 53% the one of virgin females (3.89, Fig EV3C). Similarly, we found no difference in CL c-Fos labeling across days in virgin males (Fig EV3D). Jointly, these data evidence a sex-specific engagement of the ACC-CL circuit in female mice.

Neurotransmitter identity of the ACC-CL neurocircuit

To reveal the neurotransmitter phenotype of ACC neurons engaged in the development of experience-induced parental care in virgin females, we performed *in situ* hybridization for *cfos* with markers of excitatory (*Slc17a7*/*Vglut1*) and inhibitory (*Gad1*/*Gad67*) neurons after days 1 and 3 of pup retrieval (Fig EV3E and F). We observed a significant change in the population of *Slc17a7*$^+$ neurons expressing *cfos* across testing days (P = 0.008), whereas the contingent of *Gad1*$^+$ cells co-expressing *cfos* remained unchanged (Fig EV3G). Thus, we suggest that excitatory output from the ACC could coincide with the onset of the behavioral display.

Next, we asked if thalamic inputs to the ACC could preferentially terminate on excitatory ACC neurons. Therefore, we labeled thalamic inputs using AAV vectors driving the expression of the TVA-2A-N2cG cassette under the control of either the *Camk2* promoter (AAV-*Camk2*-TVA-2A-N2cG; excitatory) or the *Dlx* promoter (AAV-Dlx-TVA-2A-N2cG, inhibitory), alongside AAV-*Camk2*-GFP or AAV-*Dlx*-GFP serving as respective negative controls (Fig EV4A and B). Two weeks later, $\text{RVdG}_{\text{envA}}$-CVS-N2c-tdTomato vectors were injected into the same region, leading to retrograde labeling of thalamic projections to ACC excitatory/inhibitory neurons (Fig 3A–C). Quantification of tdTomato$^+$ cells in the CL, normalized by the total thalamic count of tdTomato$^+$ cells revealed no difference in the density of CL projections to excitatory (4.2 ± 2.08%) and inhibitory (3.3 ± 1.03%) neurons in ACC ($n = 3/FM_{\text{pups}}$, $n = 4/V_{\text{pups}}$, $n = 4/V_{\text{obj}}$; Fig 3D), indicating no predefined anatomical bias of CL neurons to targeting either the excitatory or inhibitory subpopulation of neurons within the ACC. However, when we determined the functional ACC-CL circuit architecture of acquired maternal behavior, we observed an increase in the density of active CL inputs to ACC excitatory neurons (20.2 ± 1.3%) relative to those terminating on inhibitory ACC neurons (7.7 ± 4.8%; $n = 3/FM_{\text{pups}}$, $n = 4/V_{\text{pups}}$, $n = 4/V_{\text{obj}}$; P = 0.06; Fig 3D). Together, these data suggest that excitatory neurons of the CL preferentially activate excitatory ACC neurons, thus forming an excitatory microcircuit in which feed-forward/recurrent excitation seems essential for the acquisition of maternal behavior in virgin females.

Neuropeptide identity of CL neurons

We also examined if the population of CL neurons projecting to the ACC could be defined by a neuropeptide mark. To this end, we tested for galanin (*Gal*), as *Gal*$^+$ neurons predominantly populate the CL, PCN, and CM (Pérez *et al*, 2001) (Fig 3E) and other contingents of *Gal*$^+$ neurons drive hypothalamic circuits of parental care and other social behaviors (Wu *et al*, 2014; Kohl & Dulac, 2018; preprint: Tripp *et al*, 2019). We first evaluated whether *Gal*$^+$ neurons in CM, PCN, and CL are activated during the 1st day of the pup retrieval test, in comparison to the Gal-negative (*Gal*$^-$) neurons in these thalamic regions. Immunohistochemistry for c-Fos in Gal-Cre::FLEX-YFP female mice after pup retrieval showed a selective recruitment of *Gal*$^+$ neurons over *Gal*$^-$ cells (ratio = 5.6) in the CL, but not in the PCN (ratio = 0.98) or the CM (ratio = 0.44) (n = 3/group; CL vs. CM P = 0.04; CL vs. PCN P > 0.05; Fig 3F and G). No difference was found following object exposure ($n = 3/V_{pups}$, $n = 2/V_{obj}$; Fig 3G), suggesting a specific participation of CL^{Gal} neurons in the initiation of maternal behavior in virgin females.

We next explored the extended network of *Gal*$^+$ neurons in the CL region. For this purpose, we performed dual anterograde and retrograde labeling by combining Cre-dependent AAV-GFP and $\text{RVdG}_{\text{envA}}$-CVS-N2c-tdTomato vectors from CL of Gal-Cre mice (Figs 4A and EV4C and D). While the ACC was shown to receive input from diverse brain regions, tracing experiments from CL^{Gal} neurons demonstrated dominant projections from the ACC (65%), with sparser input from the retrosplenial area (RSP, 16%), PAG (12%), and zona incerta (ZI, 7%; Fig 4B–D). Moreover, axon tracing confirmed that CL^{Gal} neurons project to the ACC, indicating reciprocal connectivity between this cell population and ACC projection neurons (Fig 4C).

Chemogenetic manipulation of ACC neurons biases pup retrieval in virgin females

We next deployed a chemogenetic approach to selectively manipulate the activity of ACC neurons to justify the role of ACC in acquired maternal behavior in virgin females. AAV vectors

© 2022 The Authors

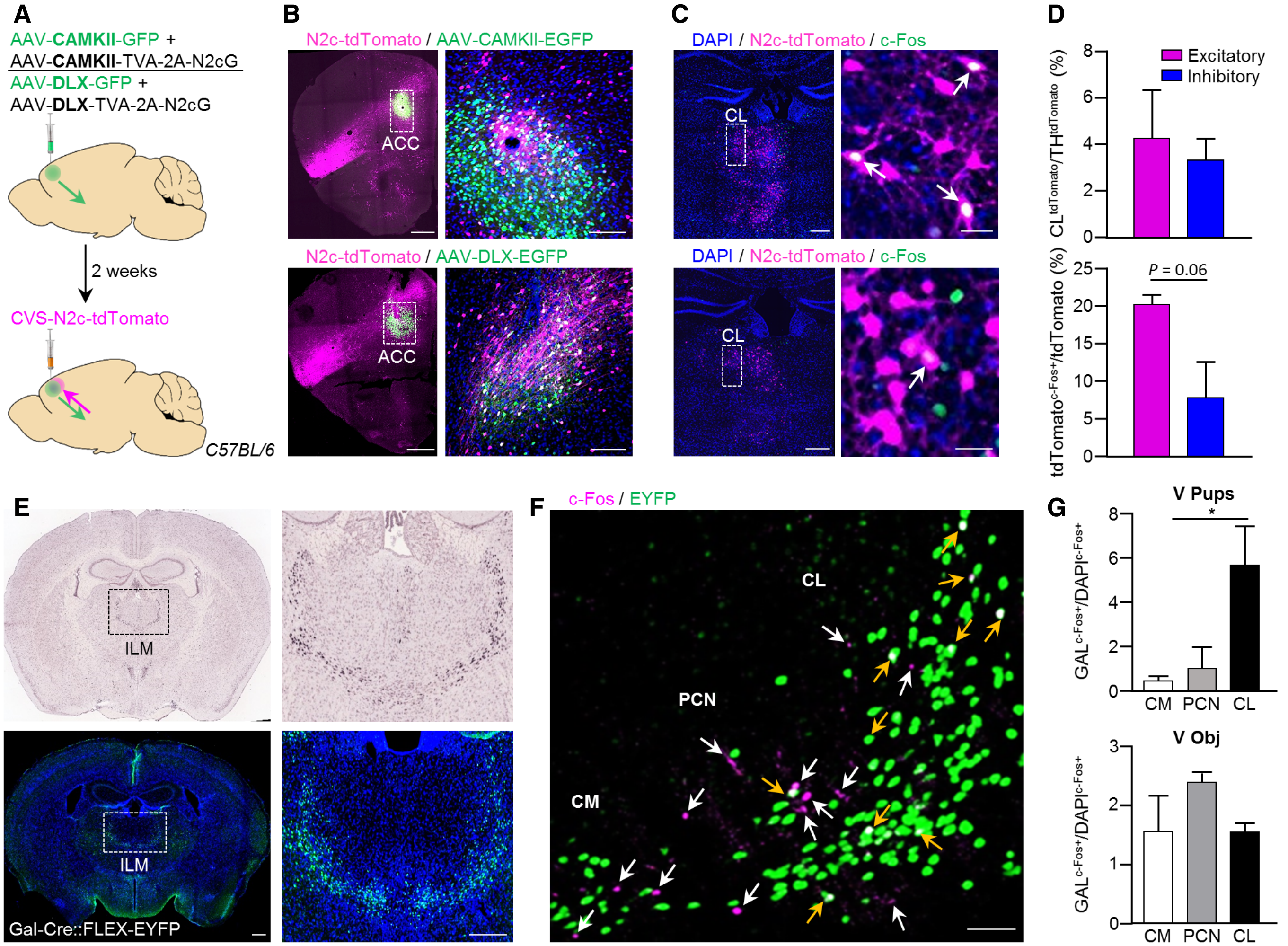

Figure 3. Functional and molecular properties of the CL-ACC projection.

A Schematic representation of the dual anterograde and retrograde tracing scheme for quantification of differential thalamic input to excitatory/inhibitory neurons in ACC.

B Representative coronal sections of the PFC, following the labeling schemes described in (A), for mapping inputs onto ACC excitatory (B, top) and inhibitory (B, bottom) neurons. High-magnification images (B, right top and bottom) show the double-labeled, putative starter cells for transsynaptic retrograde labeling. Scale bars represent 200 μm, 10× magnification (left top and bottom), and 50 μm (right top and bottom), 20× magnification.

C Representative coronal images of the thalamus, immunolabeled for c-Fos, following retrograde labeling from excitatory (C, top) or inhibitory (C, bottom) neurons in the ACC. White arrows in high-magnification images indicate co-localization of c-Fos and td-Tomato (C, right top and bottom). Scale bars represent 200 μm, 10× magnification (left top and bottom), and 25 μm (right top and bottom), 20× magnification.

D Quantification of the number of neurons in CL region projecting to excitatory or inhibitory ACC neurons, as a fraction of the total thalamic contribution (top) and a fraction of neurons co-expressing tdTomato and c-Fos of the total $tdTomato^{+}$ population in CL (bottom). Foster mothers (n = 3), virgins with pups (n = 4), and virgins with object (n = 4). Data were analyzed using Student's t-test and are displayed as mean ± s.e.m.

E Representative *in situ* hybridization images of *Gal* in the ILM taken from the Allen Mouse Brain Atlas, https://mouse.brain-map.org/experiment/ivt?id=70231997 (top left and right) and representative coronal images showing the expression of Gal neurons in Gal-Cre::FLEX-YFP female mice (bottom right and left). Scale bars represent 500 μm, 10× magnification (left bottom) and 200 μm (right bottom).

F Representative image of the ILM immunolabeled for GFP and c-Fos, following pup retrieval test. Yellow and white arrows indicate c-Fos-expressing Gal-positive and Gal-negative neurons, respectively. Scale bars represent 100 μm, 20× magnification.

G Graph plots showing the fraction of c-Fos-expressing Gal-positive neurons in the CL, PCN, and CM after pup retrieval test (G, top, n = 3) or object exposure (G, bottom, n = 2). Data were analyzed using two-way ANOVA and expressed as mean ± s.e.m.; *$P < 0.05$.

Source data are available online for this figure.

conditionally expressing hM3D or hM4D chemogenetic actuators, as well as an inert fluorophore (GFP; as control) were bilaterally injected into the ACC of virgin female mice. CNO (3 mg/kg; i.p.) or vehicle (saline) was administrated 15 min prior to the pup retrieval test, which was conducted 3 weeks after the surgery (Fig 5A). CNO applied to GFP-injected mice was used to control for possible effects of CNO alone (n = 8). Virgin females expressing hM3D in ACC showed significantly shorter latencies to retrieve pups once exposed

 © 2022 The Authors

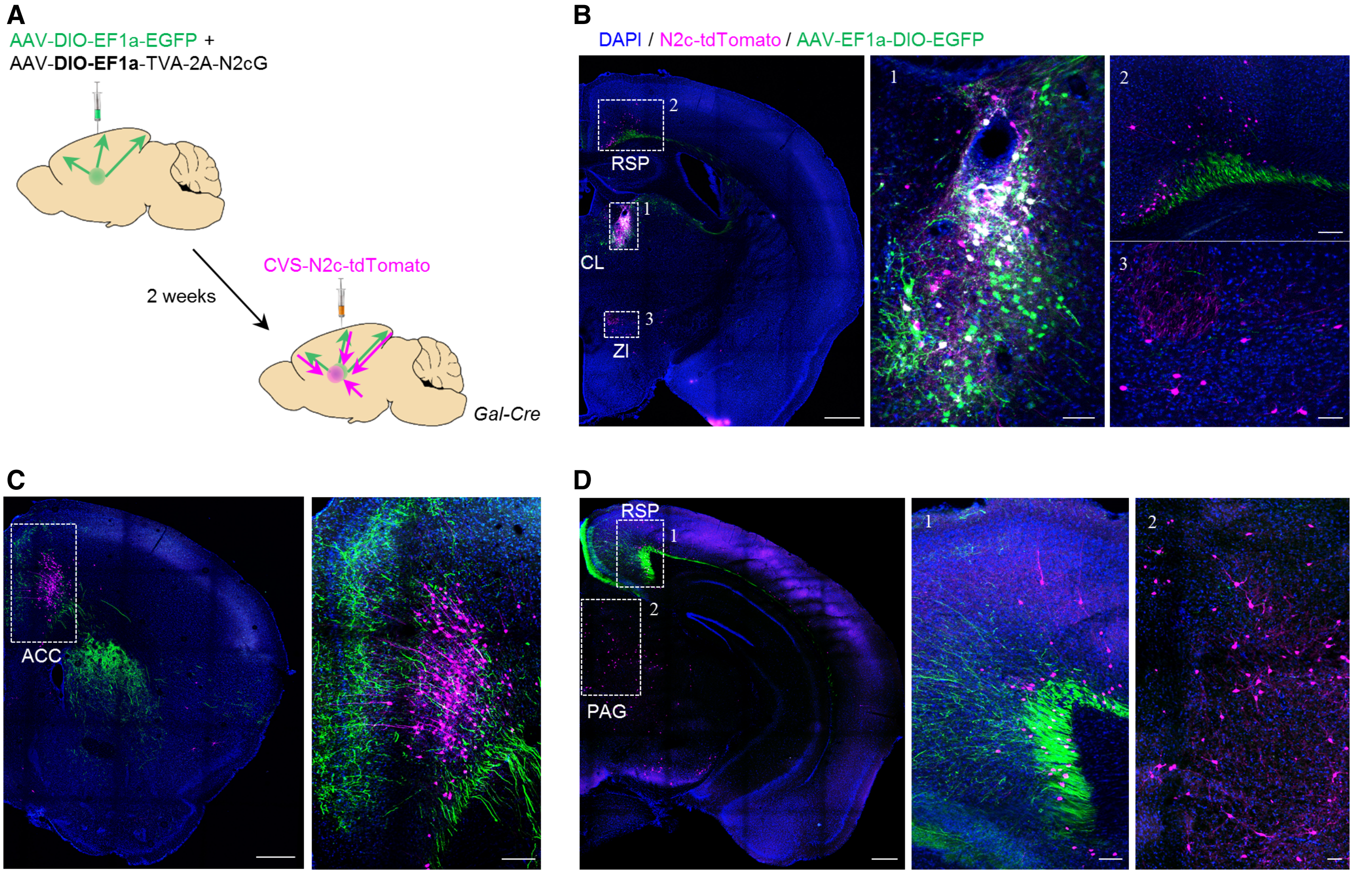

Figure 4. Tracing of CL^{Gal} neuronal connectivity.

A Schematic representation of the dual anterograde and retrograde tracing experiments from and to CL^{Gal} neurons.

B Representative images demonstrating specific targeting of the CL in Gal-Cre mice, for dual anterograde (green) and retrograde (magenta) tracing. Scale bars represent 200 μm, 10× magnification. Expanded images to the right show the CL^{Gal} neurons (B-1) and monosynaptic input neurons in the anterior RSP (B-2) and ZI (B-3). Scale bars represent 100 μm, 20× magnification.

C, D Representative images showing bidirectional connectivity with CL^{Gal} in the ACC (C) and the posterior RSP (D-1) and input from the PAG (D-2). Scale bars represent 200 μm, 10× magnification (left), and 100 μm, 20× magnification (middle and right, 1,2).

to CNO on the 1st day of pup exposure, as compared to GFP or vehicle administration (n = 6; hM3D + CNO vs. hM3D − CNO P = 0.03; hM3D + CNO vs. GFP + CNO P = 0.02; Fig 5B). The opposite effect was observed after hM4D-mediated ACC inhibition, where virgin females showed significant longer latencies to retrieve pups, as compared to GFP or vehicle administration (n = 8; hM4D + CNO vs. GFP + CNO P = 0.002; hM4D + CNO vs. hM4D − CNO P = 0.001; Fig 5C).

To address if the ACC is specifically important for pup retrieval behavior in virgins, we also evaluated the response of biological mothers to the chemogenetic manipulation of ACC activity. Since mothers retrieve the pups significantly faster than virgin females on the 1st day, with little room for further enhancement in their performance, we focused on the effect of the chemogenetic inhibitor hM4D. Neuronal inhibition in the ACC had no effect on pup retrieval behavior in mothers (n = 7; Fig 5D), suggesting selective recruitment of the ACC when being in the process of acquiring maternal behavior for the first time. Indeed, no effect of hM4D on pup retrieval latencies was observed when CNO was administrated to virgin females on the 2nd day of pup exposure (n = 5; Fig EV5A and B), further corroborating the specific engagement of the ACC in the initial triggering of maternal behavior upon first-time pup exposure.

To control for potential unspecific effects of the chemogenetic actuators on locomotion, which could bias the performance in the pup retrieval test, we quantified the total distance traveled in the Open Field Test 15 min after CNO administration in a separate cohort of virgin females. No significant difference was found between hM3D/hM4D-injected mice, as compared to GFP groups (n = 5/group; Fig EV5C and D).

To further justify the effect of hM3D on neuronal activation in the ACC-CL circuitry, c-Fos expression was quantified in the site of injection in the ACC (Fig 5E and F), as well as in the CL of virgin female mice treated with vehicle or CNO (Fig 5G). For this, we first calculated c-Fos density in the ACC as a fraction of c-Fos^+/hMD3-transduced cells and c-Fos^+/$DAPI^+$ cells, over the total $hMD3^+$/DAPI cell count. Mice treated with CNO showed a significant increase in neuronal activation in the total ACC area (P = 0.03) and in

© 2022 The Authors

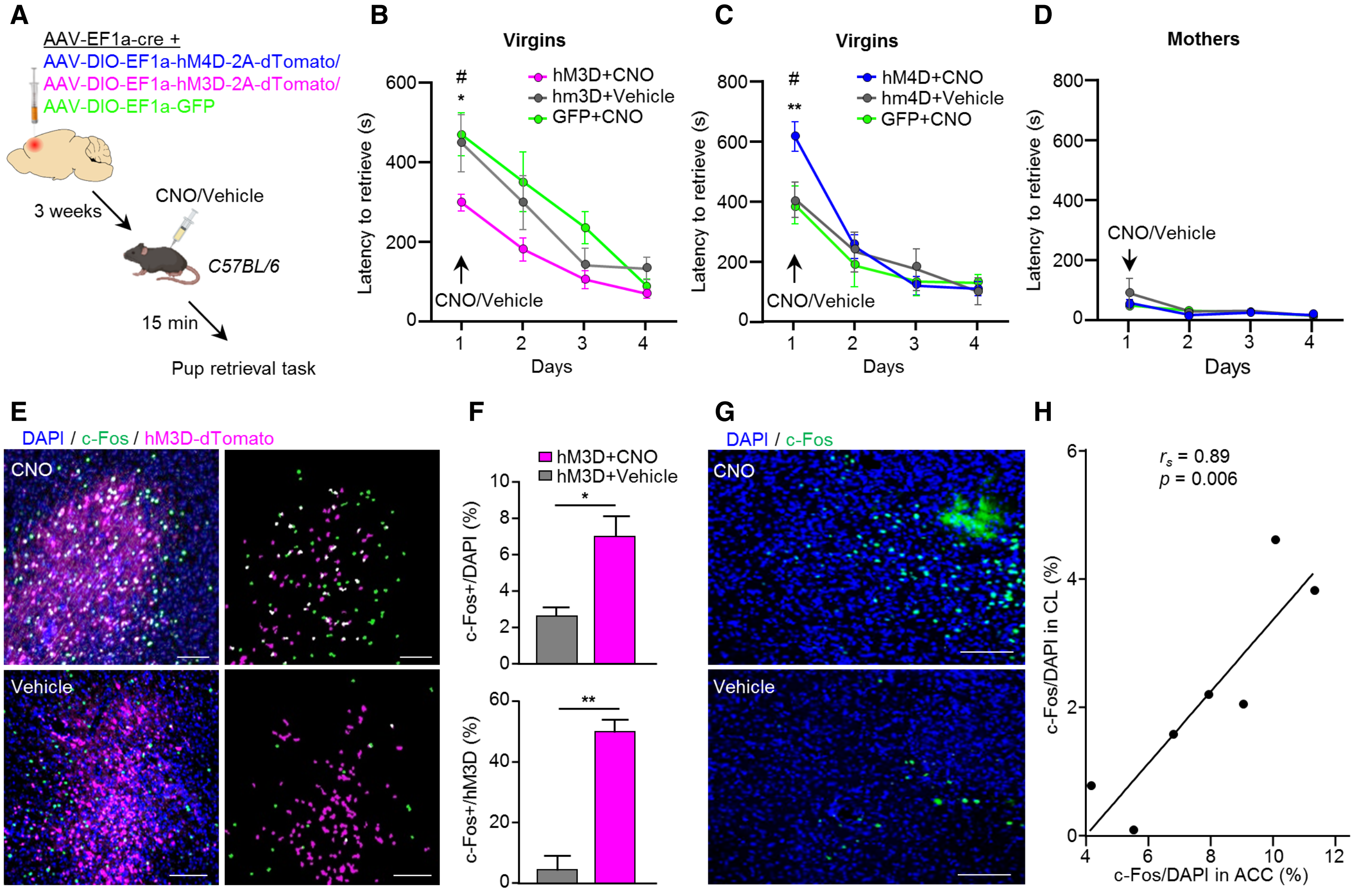

Figure 5. Acute chemogenetic activation/inhibition of the ACC affects the display of maternal care behavior in virgin females.

A Schematic representation of the chemogenetic manipulation strategy in C57BL/6 mice.

B, C Quantification of pup retrieval latencies following ACC chemogenetic activation with hM3D (n = 6) (B) or chemogenetic inhibition using hM4D (n = 7) (C) compared to control groups in virgin females (n = 8). Data were analyzed using two-way ANOVA with repeated measures and are displayed as mean ± s.e.m.; *$P < 0.05$, **$P < 0.01$. *significantly different between groups on day 1. #significantly different between days within groups, $P < 0.05$.

D Same as (C) but for mothers (n = 7).

E Representative images showing AAV-DIO-CAG-hM3D-2A-tdTomato and c-Fos expression in the PFC of virgin females, 2 h following CNO/vehicle administration. Masks demonstrating distribution of DAPI + c-Fos (green), DAPI + dTomato (magenta), and their co-localization (white). Scale bars 100 μm, 20× magnification.

F Fraction c-Fos$^+$ cells of the total DAPI (top) or hM3D-2A-dTomato (bottom) population in the ACC, following CNO/vehicle administration (n = 3). Data were analyzed using Student's t-test and are displayed as mean ± s.e.m.; *$P < 0.05$, **$P < 0.01$.

G Representative images of the CL, immunolabeled for c-Fos, following hM3D-mediated neuronal activation of the ACC. Scale bars 200 μm, 20× magnification.

H Spearman correlation between hM3D-mediated activation of the ACC (as measured in (F, top)) and the CL region.

Source data are available online for this figure.

transduced neurons (P = 0.0002), as compared to vehicle (n = 3; Fig 5F). These data confirm that hM3D-mediated excitation indeed triggered neuronal activity in the ACC. Moreover, a significant positive correlation (r_s = 0.89, P = 0.006) between c-Fos labeling in the ACC and CL was found (Fig 5H), reinforcing the functional interplay of these two brain regions.

Next, we tested whether the specific activation of Gal$^+$ neurons in the CL (CL^{Gal+}) is sufficient to drive pup retrieval in virgin females. Therefore, we bilaterally expressed hM3D or GFP in the CL of Gal-Cre virgin females (Fig 6A). We confirmed that CNO administration to hM3D-injected animals led to the selective activation of CL^{Gal+} neurons by using an increase in c-Fos expression as a surrogate (Fig 6B). Chemogenetic activation of CL^{Gal+} neurons in virgin females during the 1st day of pup exposure resulted in significantly shorter latencies to retrieve pups, as compared to GFP-expressing controls (n = 7; P = 0.02; Fig 6C). In sum, our results demonstrate that the engagement of the ACC-CL^{Gal} circuit is sufficient to trigger the display of maternal behavior in virgin females. Thereby, we can distinguish acquired from instinctive maternal care behavior at the neural circuit level.

Discussion

Parental behavior is a highly conserved, yet dynamic behavioral display (Bukhari et al, 2019). On the one hand, a decrease in the

 © 2022 The Authors

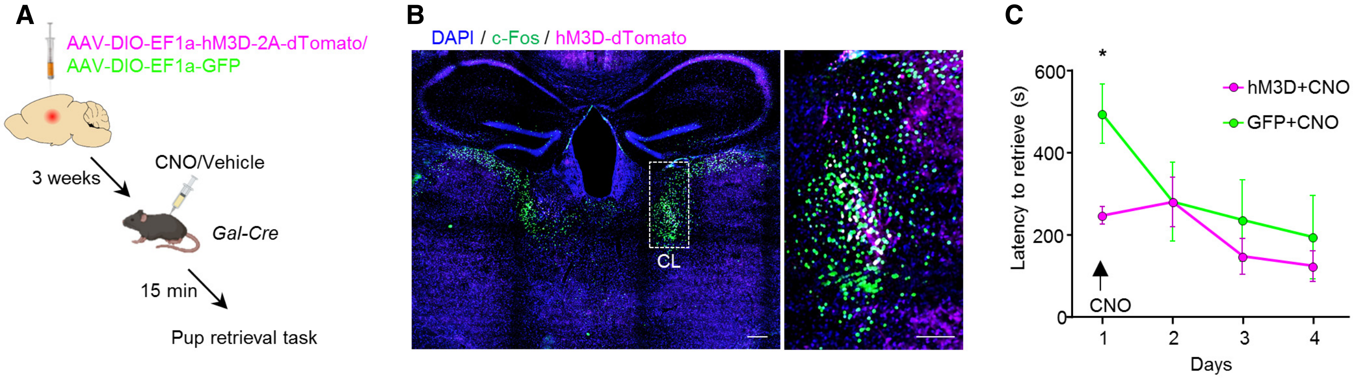

Figure 6. Acute chemogenetic activation of CL^{Gal+} neurons reduces pup retrieval latencies in virgin female mice.

A Schematic representation of the chemogenetic manipulation strategy in Gal-Cre mice.

B Representative images showing AAV-DIO-CAG-hM3D-2A-tdTomato and c-Fos expression in the CL, 2 h after CNO administration. Scale bars 200 μm, 10× magnification (left), and 50 μm, 20× magnification (right).

C Quantification of pup retrieval latencies in virgin females following CL^{Gal+} chemogenetic activation with hM3D compared to GFP-expressing control group (n = 7). Data were analyzed using two-way ANOVA with repeated measures and are presented as mean ± s.e.m; *$P < 0.05$.

Source data are available online for this figure.

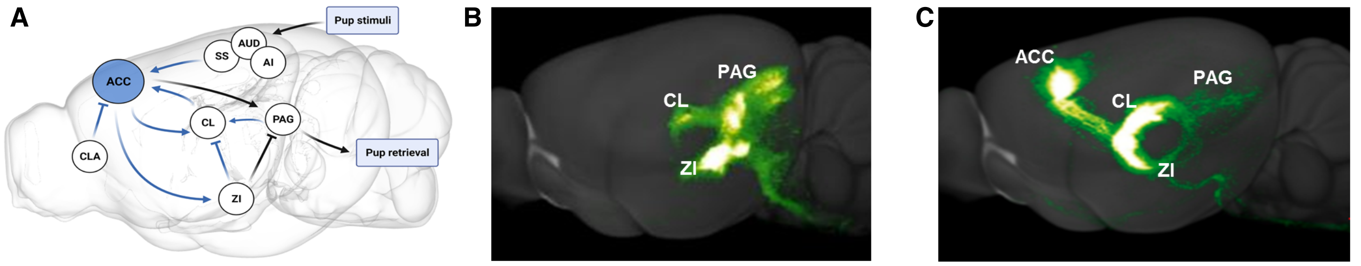

Figure 7. Model for the neural circuit underlying the acquisition of maternal behavior in virgin female mice.

A Schematic illustration of the proposed circuit model underlying the acquisition of maternal behavior in virgin females.

B, C Whole brain rendering of projections from inhibitory ZI (Slc32a1-IRES-Cre; B) and ACC layer 6 (Syt6-Cre mouse; C) outputs showing projections from both regions to the PAG, which provide further anatomical support for the suggested model. Images were taken from Allen Mouse Brain Connectivity Atlas (ZI: https://connectivity.brain-map.org/projection/experiment/171065906 and ACC: https://connectivity.brain-map.org/projection/experiment/299829892).

drive to tend to, and protect the offspring occurs under pathological conditions, such as in mothers suffering from postpartum depression or postpartum psychosis (Ghaedrahmati *et al*, 2017; Işık *et al*, 2017). On the other hand, parental care behavior can also increase, such as by exposure to infant cues in previously naive virgin rodent females (Stolzenberg & Rissman, 2011; Stolzenberg & Mayer, 2019). While the neuronal circuitry of instinctive maternal behavior, promptly exhibited by postpartum females after delivery, has been substantially explored (Dulac *et al*, 2014; Wu *et al*, 2014; Kohl *et al*, 2015, 2017; Kohl & Dulac, 2018), the neurocircuit underlying the acquisition of parenting upon pup exposure remains unknown.

Significant evidence from studies in experimental animals and humans suggests that the prefrontal cortex is a central hub orchestrating social behaviors (Yizhar & Levy, 2021). The gradual reduction in ACC activation along days of pup exposure reinforces the concept of functional adaptability of the prefrontal cortex, supporting its role in behavioral flexibility (Murray *et al*, 2015). This view also integrates our observation that the chemogenetic enhancement of ACC function presumably allows for a more rapid adaptation to a novel social environment, and thus primes a more effective selection of an appropriate behavioral strategy. The finding that chemogenetic stimulation of the ACC only affects the behavioral performance of virgin females when delivered on day 1 substantiates that the ACC-CL loop is specifically recruited to facilitate the behavioral response upon first-time pup exposure.

The CL is one of the higher-order thalamic nuclei, which forms recurrent excitatory connections with the cortex to modulate the efficacy of synaptic transmission according to behavioral demands (Stolzenberg & Rissman, 2011; Saalmann, 2014). Within the CL, we identified *Gal*$^{+}$ neurons to be specifically relevant for the initial onset of maternal behavior in virgin females. We demonstrate reciprocal connections of CL^{Gal} neurons with the ACC, which constitutes a monosynaptic excitatory reinforcement loop whose activity is necessary to trigger the initial acquisition of maternal behavior and chemogenetically validate the cell-type specificity of the ACC-CL in

© 2022 The Authors

the *Gal*-cre mouse system. Unexpectedly, our circuit mapping did not reveal a direct synaptic efferent pathway from either the ACC or CL to the mPOA. Therefore, we suggest that signal integration instead occurs at the level of the PAG, which is directly innervated by the ACC (Fig 7C), as well as the ZI (Fig 7B), projects to CL^{Gal} neurons (Fig 4C), and is potentially responsible for the execution of the behavioral end-point (Kohl *et al*, 2017) (i.e., pup retrieval; Fig 6A–C). Alternatively, upstream control could be exerted by inhibitory afferents of either the claustrum (Jackson *et al*, 2018; Narikiyo *et al*, 2020) or ZI (Barthó *et al*, 2007; Wang & Chou, 2020) to suppress ACC activity (Fig 7A).

Overall, our data describe a neurocircuit, which is essential for virgin female mice to engage in caring for newborns. We propose that recruitment of this accessory circuit allows performing maternal care, even when pregnancy and parturition-related hormonal influences cannot act on the female brain by facilitating the recruitment of downstream effector regions of the maternal care circuit upon first-time pup contact. In a wider context, this implies that alternative neural routes could exist to tune the output of the circuitry when mPOA-centered core activation is insufficient. An initial trigger to this pathway then allows for the subsequent, experience-dependent enhancement of behavioral performance.

Thus, our observation that positive parental displays can be learnt, and the delineation of the underlying circuit mechanisms could bear relevance to, e.g., postpartum depression, where the therapeutic goal is to improve mother–child interactions, supporting both the well-being of the mother and the development of the child.

Materials and Methods

Animals and housing

Wild-type C57BL/6J mice were purchased from Charles River (Sulzfeld, Germany). Reporter strains FLEX-tdTomato (Gt(Rosa)26Sortm14(CAGtdTomato), JAX mice stock # 007914) and FLEX-YFP (B6.129X1-Gt(ROSA)26Sortm1(EYFP)Cos/J, JAX mice stock # 006148) have been imported from Jackson Laboratories. The Gal-Cre line (Tg(Gal-cre)KI87Gsat/Mmucd, 031060-UCD) was obtained from Mutant Mouse Resource & Research Centers (MMRRC). All animals were maintained on 12 h:12 h light/dark cycle with food and water available *ad libitum*. Experiments on mice were approved by the Federal Ministry of Education, Science and Research of Austria (2021-0.744.367), and conducted to minimize animal suffering and keep the needed animal numbers at a minimum level.

Behavioral assays

Mice (8–12 weeks at experiment onset) were single housed in standard cages for a period of 7–14 days before the onset of experiments. All behavioral tests were conducted during the light phase. Behaviors were recorded using multi-camera surveillance (Axis IP cameras-Noldus, Wageningen, Netherlands) and analyzed using Observer XT or EthoVision software (Noldus, Information Technology, Wageningen, Netherlands) by an experimenter blinded to the experimental groups.

Pup retrieval by females

Three randomly selected pups (day of birth: 0–2) provided by a donor mother (for foster mother and virgin groups) or taken from the tested animal's litter (for biological mother group) were individually placed into the resident mouse home cage on the side opposite to the nest. Virgin female mice were presented with either 3 foster pups or 3 novel objects (Lego® blocks, 1.6 cm × 1.1 cm). Retrieving behavior was measured as the latency until the female retrieved all 3 pups back into the nest, with a maximum time of 15 min assay (Kohl & Dulac, 2018; Scott *et al*, 2015). Within this period, we also monitored the total duration of pup-directed (crouching, licking and grooming, and covering over the pups) and non-pup-directed behaviors (including self-grooming, eating, and sleeping) in virgin females. Other elements related to parental care, but not involving physical interaction with the pups (e.g., nest building), were not considered. The behavioral tests were conducted for 3 consecutive days in the females' home cage.

Parenting behavior in males

Virgin male mice were presented with 3 pups placed individually in a small (round, 4.5 cm diameter) container with a metal grid (#X001I39B4R, Nuoshen, Shanghai, China) to prevent the males from attacking and killing the pups. Animals were habituated to the empty containers 24 h prior to testing. Male mice were exposed to pups for 15 min on 3 consecutive days.

Open field

The Open Field Test was performed using a computational tracking system (MedAssociates Activity Monitor, Fairfax, USA) in an arena (27.5 cm × 27.5 cm; with 21 cm high walls). The assessment of locomotor activity was based on the quantification of the total distance traveled for a 15 min period (Reisinger *et al*, 2020).

Estrous cycle staging

The stage of the estrous cycle was determined by visual inspection (assessment of the vaginal opening) prior to behavioral testing by Byers *et al*. (2012). Additionally, the estrous cycle stage was confirmed by microscopic examination of vaginal smears stained with Cresyl violet (Gage *et al*, 2020) (#26671-1A, Electron Microscopy Sciences, Hatfield, USA). The stage of the cycle was defined based on the presence or absence of leukocytes, cornified epithelial, and nucleated epithelial cells (Pu *et al*, 2015).

RNA *in situ* hybridization (ISH)

Female mice were euthanized 30 min after the pup retrieval test and brains were embedded in OCT (Tissue-Tek), frozen with dry ice, and sliced at 16 μm sections with a cryostat (Leica CM1950, Leica Mikrosysteme Handelsges.m.b.H., Vienna, Austria). First, sections were fixated with 4% PFA for 15 min, washed with PBS, and dehydrated using an ascending EtOH gradient (25, 50, 75, and 100%, each step for 5 min with subsequent drying for 15 min) (Romanov *et al*, 2020). The slices were then stained according to the manufacturer's protocol (Molecular Instruments). ISH probes for detection of *fos* (#NM_010234.3), *Slc17a7* (#NM_182993), and *gad1* (#NM_008077.5) were designed commercially by the manufacturer

 © 2022 The Authors

(Molecular Instruments). Imaging was performed using an A1 laser-scanning microscope (Nikon) with a 20× objective.

Production of viral vectors

Adeno-associated virus (AAV) production was performed in HEK293T cells based on a previously published protocol (Mcclure *et al*, 2011). Briefly, fully confluent HEK293 cells were transfected with an AAV2 vector plasmid along with pAdenoHelper and the AAV-dj RepCap plasmids using PEI. Thirty-six hours post-transfection, the cells were harvested, pelleted, and lysed using three freeze–thaw cycles. The lysed cells were incubated with benzonase nuclease (Sigma Aldrich) for 1 h and then the debris was pelleted, and the virus-containing supernatant collected and passed through a 0.22 μm filter. The collected supernatant was subsequently mixed with an equal amount of Heparin agarose (Sigma Aldrich) and kept at 4°C overnight with constant agitation. The following day, the agarose–virus mixture was transferred to a chromatography column and the agarose was allowed to settle. The supernatant was then drained from the column by means of gravity and the agarose-bound virus was washed once with PBS and then eluted using PBS supplemented with 0.5 M NaCl. The eluted virus was then filtered again, desalinated, and concentrated using a 100 kDa centrifugal filter and then aliquoted and stored at −80°C until use.

RVdG-CVS-N2c viral vectors were produced using HEK293-GT, BHK-eT, and HEK-TVA cells for rescue, pseudotyping, and titration of viral particles, respectively, according to a previously published method (Sumser *et al*, 2022). Briefly, HEK293-GT cells were transfected with the rabies vector plasmid and the SADB19 helper plasmids pTIT-N, pTIT-P, and pTIT-L using polyethyleneimine (PEI). Twenty-four hours later, the transfected cells were resuspended and re-plated in a 100 mm culture dish and incubated at 37°C/5% CO2 until they regained full confluence. Five to six days from the time of transfection, the medium was harvested, filtered, and used to transduce BHK-eT cells for simultaneous pseudotyping and amplification. Medium collected from these plates was collected daily over 3 consecutive days, pooled, and centrifuged at 70,000 rcf for 1.5 h. Following centrifugation, the medium was aspirated, and the viral pellet was resuspended in 200 μl phosphate-buffered saline (PBS), pH 7.4, aliquoted, and stored at −80°C until use.

Surgical procedures

Female mice at 8–12 weeks of age were anesthetized with 2–4% Isoflurane (Forane, AbbVie Inc.), placed in a stereotaxic frame (RWD Life Science, Shenzhen, China), and kept under constant anesthesia with oxygen at a flow rate of 800 ml/min. Viruses were injected into the ACC (coordinates: anterior–posterior (AP) 1.8 mm, medio-lateral (ML) 0.5 mm, and dorso-ventral (DV) 1.25 mm) or CL (coordinates: AP -1.2 mm, ML 0.5 mm, and DV 2.7 mm). AAV vectors were delivered to the injection site at a volume of 0.3 μl and a rate of 0.1 μl per minute, using a Hamilton syringe. At the end of the injection, the needle was left in place for 3 additional minutes to prevent the efflux of virus during the removal of the needle. The needle was then slowly removed, and the scalp was sutured. For monosynaptic retrograde tracing, AAV vectors conditionally expressing a cassette for co-expression of the TVA receptor and the rabies N2c glycoprotein (N2cG) were injected unilaterally in Gal-Cre, FLEX-tdTomato, or C57BL/6J female mice, alongside vectors expressing Cre recombinase (for FLEX-tdTomato and C57BL/6J mice). Two weeks later, pseudotyped rabies viral vectors ($RVdG_{envA}$-CVS-N2c-EGFP/tdTomato, ~2–5 × 10^8 TU/ml) were injected close to the area of the prior injection, and mice were anesthetized and perfused 5–7 days later (Sumser *et al*, 2022).

Drug preparation

Clozapine-N-oxide (CNO) (Tocris Bioscience) was first dissolved in dimethyl sulfoxide (DMSO, final concentration of 0.5%) and gently vortexed to obtain suspension (Farrell *et al*, 2013). After the solution turned translucent, 0.9% saline was added to a final concentration of 0.1 mg/ml.

Chemogenetics

Cre-dependent AAV vectors expressing the pharmacogenetic actuator hM3Dq-dTomato, hM4Di-dTomato, or EGFP alone were bilaterally injected into the ACC of C57BL/6J female mice, alongside AAVs expressing Cre recombinase. For thalamic injections, Cre-dependent AAVs (hM3Dq-dTomato or EGFP) were injected into the CL of Gal-Cre mice (RRID:MMRRC_031060-UCD). Following recovery, females were kept single housed (for testing of virgin females) or placed in a cage with a male until a vaginal plug was visible, within 1 week at the latest (for testing of mothers). Three weeks later, CNO (3 mg/kg) or vehicle (saline) was administrated 15 min before the pup retrieval test. Parental behavior was also evaluated on 2 consecutive days (day 2 and 3) without CNO administration. To further evaluate the effect of the pharmacogenetic manipulation on neuronal activity patterns, animals were sacrificed 90 min after CNO/vehicle administration, and c-Fos immunohistochemistry was conducted.

Tissue preparation and immunohistochemistry

For the evaluation of neuronal activity patterns, mice were perfused 90 min after behavioral testing (day 1/day 3). Mice were anesthetized (ketamine 100 mg/kg; xylazine: 40 mg/kg; i.p., both 10 ml/kg) and perfused transcardially with phosphate-buffered saline (PBS) followed by 4% paraformaldehyde (PFA) in PBS. Fixed brains were incubated in 30% sucrose for 48 h, frozen, embedded in OCT, and cut into free-floating 30–40 μm slices on a cryostat (Leica, Germany). A 1-in-6 series of slices were incubated with primary antibodies for rabbit anti-phospho-c-Fos (1:2,000; Cell Signaling Technology, 5348S), chicken anti-GFP (1: 500; Abcam, ab13970), and mouse anti-NeuN (1:1,000; Chemicon/Sigma-Aldrich, MAB377). Secondary antibodies are as follow: donkey anti-rabbit Alexa 647 (1:500, Invitrogen, A32795), goat anti-chicken Alexa 488 (1:500, Invitrogen, A32931), and goat anti-mouse CF 594 (1:500, Merck, SAB4600402).

Imaging and image analysis

All images were taken using either an A1 laser scanning microscope (Nikon Instruments Inc., New York, USA) or an Axiovert 200M Fluorescence/Live cell Imaging Microscope with 10× and 20× objectives (Carl Zeiss AG; Oberkochen, Germany), under identical

© 2022 The Authors

conditions for images belonging to the same set of experiments in each instance.

For quantification of c-Fos-labeled cells after the pup retrieval task, the region of interest was selected in 3 different sections and c-Fos + DAPI-expressing cells were analyzed using FIJI/ ImageJ (Schindelin *et al*, 2012). Regions with high tissue autofluorescence could not be quantified, such as the striatum and the lateral group of the dorsal thalamus (LAT). To quantify the ACC output, we used the Allen Brain Connectivity Atlas (Oh *et al*, 2014). Three different anterograde labeling experiments performed in wild-type mice (139520203, 139426984, and 112458114) were chosen based on the exclusivity of the injection site. To calculate the output density for each thalamic group, we first summed the total signal volume for all subnuclei comprising each group, and divided it by total group area, for each experiment individually. ACC output density *z*-scores were then calculated against the average density for all brain regions included in the calculation to generate a relative density value for each. The *z*-scores displayed represent the average *z*-score of the three tracing experiments. Data for individual thalamic subnuclei, belonging to the same functionally distinct nucleus, were pooled together according to a conventional classification scheme (Allen mouse brain atlas, Table EV1, Fig EV2A). Quantification of cell numbers and channel overlap in retrograde labeling experiments was done using FIJI/ ImageJ (Schindelin *et al*, 2012) on stack images. For quantification of input densities, the number of retrogradely labeled neurons was divided by the number of nuclei ($DAPI^+$) in target regions using a custom-written script (https://github.com/sommerc/coloco3surf; Ben-Simon *et al*, 2022). ACC input density *z*-scores were then calculated against the average density for all brain regions included in the calculation to generate a relative density value for each. To determine which of the observed ACC input populations were preferentially activated during pup retrieval, the fraction of c-Fos + RVdG-CVS-N2c-tdTomato co-expressing cells, over the total RVdG-CVS-N2c-tdTomato count, was calculated. To quantify the activation of Gal^+ cells, the number of Gal-positive neurons was divided by the number of all c-Fos^+ cells in the CM, PCN, and CL nuclei.

Statistics

Sample sizes were determined on the basis of previous experiences in the laboratory and accepted practice based on published data (Ronovsky *et al*, 2017; Zhao *et al*, 2017; Liao *et al*, 2019), but no statistical analysis was used to predetermine sample size. Data were analyzed by two-tailed, unpaired Student's *t*-test; Fisher's exact test, one-way ANOVA, repeated measures one-way ANOVA, or two-way ANOVA followed by Tukey's *post hoc* test was appropriate. For correlation analysis, Spearman's rank correlation coefficient was used. All statistical tests were performed using Graph Pad Prism 9. Full statistical details are provided in the respective figure legends.

Data availability

This study includes no data deposited in external repositories.

Expanded View for this article is available online.

Acknowledgements

We thank Maureen Cabatic for technical support, Orsolya Horvath for help with animal maintenance, and Jenny Kral for image analysis, as well as other members of the Pollak laboratory for their advice. This work is supported by the Austrian Science Fund (Fonds für Wissenschaft und Forschung, FWF; grant no. I 4854 and P 34281 to DDP), ERC advanced grant (FOODFORLIFE 2020-AdG-101021016, to TH). AG is supported by the FWF doctoral program DOC 33-B27.

Author contributions

Micaela Glat: Conceptualization; formal analysis; investigation; writing – original draft. **Anna Gundacker:** Formal analysis; investigation; visualization; methodology; writing – original draft; writing – review and editing. **Laura Cuenca Rico:** Formal analysis; visualization. **Barbara Czuczu:** Investigation. **Yoav Ben-Simon:** Methodology. **Tibor Harkany:** Conceptualization; supervision; writing – original draft; writing – review and editing. **Daniela D Pollak:** Conceptualization; formal analysis; supervision; funding acquisition; writing – original draft; project administration; writing – review and editing.

Disclosure and competing interests statement

The authors declare that they have no conflict of interest.

References

Angoa-Pérez M, Kuhn DM (2015) Neuronal serotonin in the regulation of maternal behavior in rodents. *Neurotransmitter (Houst)* 2: e615

Barroso-Chinea P, Castle M, Aymerich MS, Lanciego JL (2008) Expression of vesicular glutamate transporters 1 and 2 in the cells of origin of the rat thalamostriatal pathway. *J Chem Neuroanat* 35: 101–107

Barthó P, Slézia A, Varga V, Bokor H, Pinault D, Buzsáki G, Acsády L (2007) Cortical control of zona incerta. *J Neurosci* 27: 1670–1681

Ben-Simon Y, Kaefer K, Velicky P, Csicsvari J, Danzl JG, Jonas P (2022) A direct excitatory projection from entorhinal layer 6b neurons to the hippocampus contributes to spatial coding and memory. *Nat Commun* https://doi.org/10.1038/s41467-022-32559-8

Bosch OJ (2011) Maternal nurturing is dependent on her innate anxiety: the behavioral roles of brain oxytocin and vasopressin. *Horm Behav* 59: 202–212

Brunton PJ, Russell JA (2008) The expectant brain: adapting for motherhood. *Nat Rev Neurosci* 91: 11–25

Bukhari SA, Saul MC, James N, Bensky MK, Stein LR, Trapp R, Bell AM (2019) Neurogenomic insights into paternal care and its relation to territorial aggression. *Nat Commun* 101: 1–11

Byers SL, Wiles MV, Dunn SL, Taft RA (2012) Mouse estrous cycle identification tool and images. *PLoS One* 7: 35538

Carcea I, Caraballo NL, Marlin BJ, Ooyama R, Riceberg JS, Mendoza Navarro JM, Opendak M, Diaz VE, Schuster L, Alvarado Torres MI *et al* (2021) Oxytocin neurons enable social transmission of maternal behaviour. *Nature* 596: 553–557

D'Amato FR, Scalera E, Sarli C, Moles A (2005) Pups call, mothers rush: does maternal responsiveness affect the amount of ultrasonic vocalizations in mouse pups? *Behav Genet* 35: 103–112

Dulac C, O'Connell LA, Wu Z, O'Connell LA, Wu Z, O'Connell LA, Wu Z, O'Connell LA, Wu Z (2014) Neural control of maternal and paternal behaviors. *Science* 345: 765–770

Farrell MS, Pei Y, Wan Y, Yadav PN, Daigle TL, Urban DJ, Lee HM, Sciaky N, Simmons A, Nonneman RJ *et al* (2013) A Gαs DREADD mouse for selective modulation of cAMP production in striatopallidal neurons. *Neuropsychopharmacology* 38: 854–862

 © 2022 The Authors

Franklin TB, Silva BA, Perova Z, Marrone L, Masferrer ME, Zhan Y, Kaplan A, Greetham L, Verrechia V, Halman A *et al* (2017) Prefrontal cortical control of a brainstem social behavior circuit. *Nat Neurosci* 20: 260 – 270

Gage M, Golden M, Putra M, Sharma S, Thippeswamy T (2020) Sex as a biological variable in the rat model of diisopropylfluorophosphate-induced long-term neurotoxicity. *Ann NY Acad Sci* 1479: 44 – 64

Georgescu T, Swart JM, Grattan DR, Brown RSE (2021) The prolactin family of hormones as regulators of maternal mood and behavior. *Front Glob Womens Health* 2: 767467

Ghaedrahmati M, Kazemi A, Kheirabadi G, Ebrahimi A, Bahrami M (2017) Postpartum depression risk factors: a narrative review. *J Educ Health Promot* 6: 60

Grimsley JMS, Monaghan JJM, Wenstrup JJ (2011) Development of social vocalizations in mice. *PLoS One* 6: e17460

Işık M, Yıldırım A, Boysan M, Murat D (2017) Dissociative experiences, childhood trauma and alexithymia among mothers of children with autism spectrum disorder. *Yeni Symp* 55: 7 – 10

Jackson J, Karnani MM, Zemelman BV, Burdakov D, Lee AK (2018) Inhibitory control of prefrontal cortex by the claustrum. *Neuron* 99: 1029 – 1039

Kohl J, Dulac C (2018) Neural control of parental behaviors. *Curr Opin Neurobiol* 49: 116 – 122

Kohl J, Babayan BM, Rubinstein ND, Autry AE, Marin-Rodriguez B, Kapoor V, Miyamishi K, Zweifel LS, Luo L, Uchida N *et al* (2015) Functional circuit architecture underlying parental behaviour. *Nature* 40: 1291 – 1296

Kohl J, Autry AE, Dulac C (2017) The neurobiology of parenting: a neural circuit perspective. *Bioessays* 39: 1 – 11

Kung JC, Shyu BC (2002) Potentiation of local field potentials in the anterior cingulate cortex evoked by the stimulation of the medial thalamic nuclei in rats. *Brain Res* 953: 37 – 44

Lee E, Rhim I, Lee JW, Ghim JW, Lee S, Kim E, Jung MW (2016) Enhanced neuronal activity in the medial prefrontal cortex during social approach behavior. *J Neurosci* 36: 6926 – 6936

Liao GY, Kinney CE, An JJ, Xu B (2019) TrkB-expressing neurons in the dorsomedial hypothalamus are necessary and sufficient to suppress homeostatic feeding. *Proc Natl Acad Sci USA* 116: 3256 – 3261

Martyn AC, Choleris E, Gillis DJ, Armstrong JN, Amor TR, McCluggage ARR, Turner PV, Liang G, Cai K, Lu R (2012) Luman/CREB3 recruitment factor regulates glucocorticoid receptor activity and is essential for prolactin-mediated maternal instinct. *Mol Cell Biol* 32: 5140 – 5150

Mcclure C, Cole KL, Wulff P, Klugmann M, Murray AJ (2011) Production and titering of recombinant adeno-associated viral vectors. *J Vis Exp* 57: e3348

McHenry JA, Rubinow DR, Stuber GD (2015) Maternally responsive neurons in the bed nucleus of the stria terminalis and medial preoptic area: putative circuits for regulating anxiety and reward. *Front Neuroendocrinol* 38: 65 – 72

Murray AJ, Woloszynowska-Fraser MU, Ansel-Bollepalli L, Cole KLH, Foggetti A, Crouch B, Riedel G, Wulff P (2015) Parvalbumin-positive interneurons of the prefrontal cortex support working memory and cognitive flexibility. *Sci Rep* 51: 1 – 14

Narikiyo K, Mizuguchi R, Ajima A, Shiozaki M, Hamanaka H, Johansen JP, Mori K, Yoshihara Y (2020) The claustrum coordinates cortical slow-wave activity. *Nat Neurosci* 236: 741 – 753

Noirot E (1972) Ultrasounds and maternal behavior in small rodents. *Dev Psychobiol* 5: 371 – 387

Numan M (2006) Hypothalamic neural circuits regulating maternal responsiveness toward infants. *Behav Cogn Neurosci Rev* 5: 163 – 190

Numan M, Insel T (2003) Hormonal and nonhormonal basis of maternal behavior. In *The Neurobiology of Parental Behavior*, Ball GF, Balthazart J, Nelson RJ (eds), pp 8 – 41. New York, NY: Springer

Oh SW, Harris JA, Ng L, Winslow B, Cain N, Mihalas S, Wang Q, Lau C, Kuan L, Henry AM *et al* (2014) A mesoscale connectome of the mouse brain. *Nature* 508: 207 – 214

Pérez SE, Wynick D, Steiner RA, Mufson EJ (2001) Distribution of galaninergic immunoreactivity in the brain of the mouse. *J Comp Neurol* 434: 158 – 185

Portfors CV, Perkel DJ (2014) The role of ultrasonic vocalizations in mouse communication. *Curr Opin Neurobiol* 28: 115 – 120

Pu BC, Fang L, Gao LN, Liu R, Li AZ (2015) Animal study on primary dysmenorrhoea treatment at different administration times. *Evid Based Complement Alternat Med* 2015: 367379

Reisinger SN, Bilban M, Stojanovic T, Derdak S, Yang J, Cicvaric A, Horvath O, Sideromenos S, Zambon A, Monje FJ *et al* (2020) Lmo3 deficiency in the mouse is associated with alterations in mood-related behaviors and a depression-biased amygdala transcriptome. *Psychoneuroendocrinology* 111: 104480

Romanov RA, Tretiakov EO, Kastriti ME, Zupancic M, Häring M, Korchynska S, Popadin K, Benevento M, Rebernik P, Lallemend F *et al* (2020) Molecular design of hypothalamus development. *Nature* 582: 246 – 252

Ronovsky M, Berger S, Zambon A, Reisinger SN, Horvath O, Pollak A, Lindtner C, Berger A, Pollak DD (2017) Maternal immune activation transgenerationally modulates maternal care and offspring depression-like behavior. *Brain Behav Immun* 63: 127 – 136

Rosenblatt JS (1967) Nonhormonal basis of maternal behavior in the rat. *Science* 156: 1512 – 1514

Saalmann YB (2014) Intralaminar and medial thalamic influence on cortical synchrony, information transmission and cognition. *Front Syst Neurosci* 8: 83

Schindelin J, Arganda-Carreras I, Frise E, Kaynig V, Longair M, Pietzsch T, Preibisch S, Rueden C, Saalfeld S, Schmid B *et al* (2012) Fiji: an open-source platform for biological-image analysis. *Nat Methods* 9: 676 – 682

Scott N, Prigge M, Yizhar O, Kimchi T (2015) A sexually dimorphic hypothalamic circuit controls maternal care and oxytocin secretion. *Nature* 525: 519 – 522

Stockley P, Hobson L (2016) Paternal care and litter size coevolution in mammals. *Proc Biol Sci* 283: 20160140

Stolzenberg DS, Mayer HS (2019) Experience-dependent mechanisms in the regulation of parental care. *Front Neuroendocrinol* 54: 100745

Stolzenberg DS, Rissman EF (2011) Oestrogen-independent, experience-induced maternal behaviour in female mice. *J Neuroendocrinol* 23: 345 – 354

Sumser A, Joesch M, Jonas P, Ben-Simon Y (2022) Fast, high-throughput production of improved rabies viral vectors for specific, efficient and versatile transsynaptic retrograde labeling. *ELife* https://doi.org/10.7554/elife.79848

Swain JE, Mayes LC, Leckman JF (2004) The development of parent-infant attachment through dynamic and interactive signaling loops of care and cry. *Behav Brain Sci* 27: 472 – 473

Tachikawa KS, Yoshihara Y, Kuroda KO (2013) Behavioral transition from attack to parenting in male mice: a crucial role ofthe vomeronasal system. *J Neurosci* 33: 5120 – 5126

Terkel J, Rosenblatt JS (1972) Humoral factors underlying maternal behavior at parturition: cross transfusion between freely moving rats. *J Comp Physiol Psychol* 80: 365 – 371

Tripp JA, Salas-Allende I, Makowski A, Bass AH (2019) Preoptic galanin neuron activation is specific to courtship reproductive tactic in fish with two male morphs. *bioRxiv* https://doi.org/10.1101/515452 [PREPRINT]

© 2022 The Authors

Tsuneoka Y (2019) Molecular neuroanatomy of the mouse medial preoptic area with reference to parental behavior. *Anat Sci Int* 94: 39 – 52

Tsuneoka Y, Maruyama T, Yoshida S, Nishimori K, Kato T, Numan M, Kuroda KO (2013) Functional, anatomical, and neurochemical differentiation of medial preoptic area subregions in relation to maternal behavior in the mouse. *J Comp Neurol* 521: 1633 – 1663

Wang X, Chou XL, Zhang LI, Tao HW (2020) Zona incerta: an integrative node for global behavioral modulation. *Trends Neurosci* 43: 82 – 87

Wöhr M, Schwarting RKW (2010) Activation of limbic system structures by replay of ultrasonic vocalization in rats. *Handb Behav Neurosci* 19: 113 – 124

Wöhr M, Oddi D, D'Amato FR (2010) Effect of altricial pup ultrasonic vocalization on maternal behavior. *Handb Behav Neurosci* 19: 159 – 166

Wu Z, Autry AE, Bergan JF, Watabe-Uchida M, Dulac CG (2014) Galanin neurons in the medial preoptic area govern parental behaviour. *Nature* 509: 325 – 330

Yizhar O, Levy DR (2021) The social dilemma: prefrontal control of mammalian sociability. *Curr Opin Neurobiol* 68: 67 – 75

Zhao S, Witten D, Shojaie A (2017) In defense of the indefensible: a very naive approach to high-dimensional inference. *Stat Sci* 36: 562 – 577

License: This is an open access article under the terms of the Creative Commons Attribution License, which permits use, distribution and reproduction in any medium, provided the original work is properly cited.

Expanded View Figures

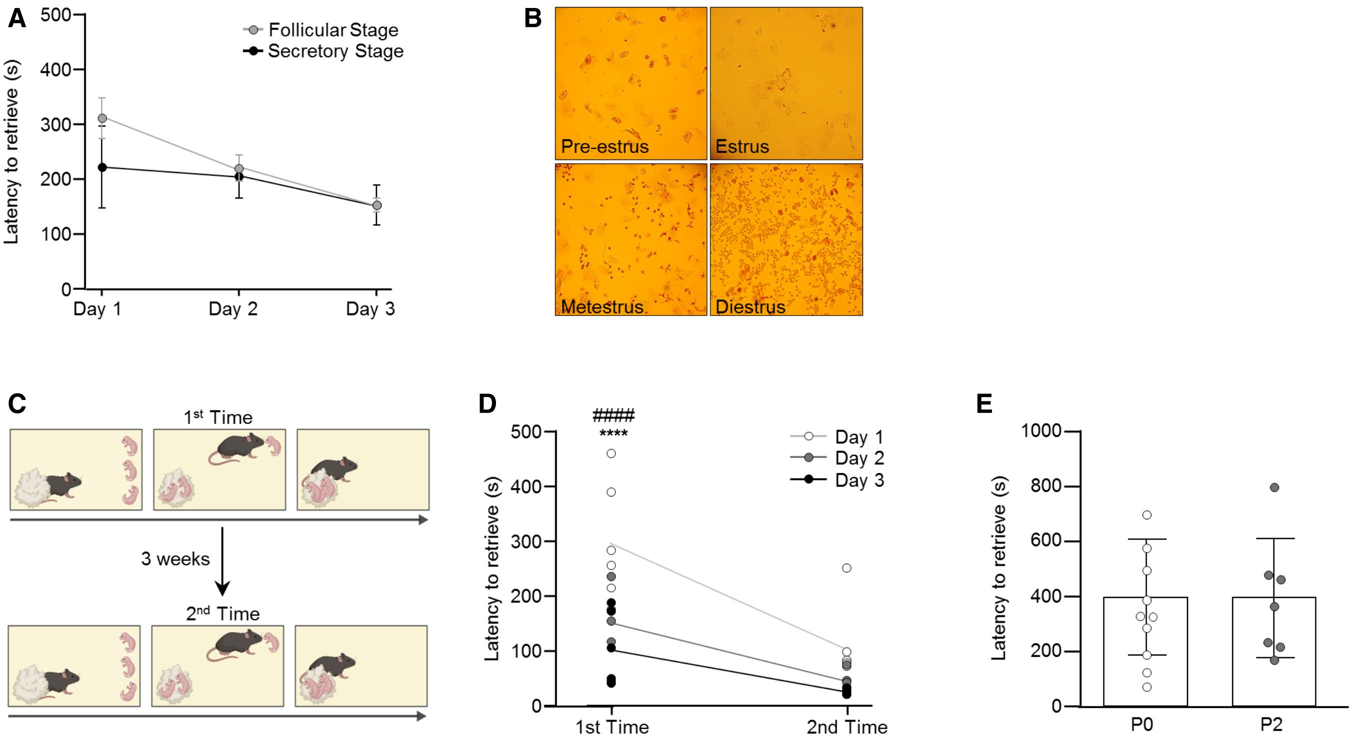

Figure EV1. Pup retrieval behavior in virgin female mice.

A Total pup retrieval time of virgin females in the follicular or secretory stage of the estrus cycle ($n = 4$/group). Data were analyzed using two-way ANOVA with repeated measures and are expressed as mean ± s.e.m.

B Representative images of vaginal smears of pre-estrus, estrus, metestrus, and diestrus stage.

C Schematic representation of the pup retrieval test at different time points.

D Total pup retrieval time in the first and second rounds of behavioral testing in virgin female mice ($n = 6$/group). Data were analyzed using two-way ANOVA with repeated measures and are expressed as mean ± s.e.m. ****significantly different from foster mothers, $P < 0.0001$; ####significantly different from mothers, $P < 0.0001$.

E Total pup retrieval time in virgin females exposed to pups at different ages (P0 and P2) on the first day of pup exposure (P0: $n = 10$: P2: $n = 7$). Data were analyzed using Student's t-test and displayed as individual values ± s.e.m.

Source data are available online for this figure.

© 2022 The Authors

Figure EV2. ACC projections to different brain structures.

A Schematic illustration of the analyzed thalamic substructures.

B, C Heat-maps of the *z*-scores based on the average ACC output density to the different thalamic (B) and hypothalamic (C) structures.

A

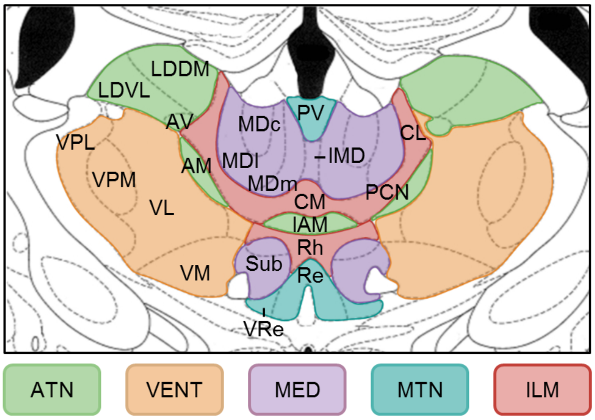

B

Region	Structure	Substructure	Z-score
TH	ATN	AM	2.07
		LD	1.34
		IAD	1.22
		IAM	0.08
		AV	-0.05
		AD	-1.1
	ILM	CL	2.06
		PF	-0.14
		RH	-0.53
		PCN	-1.17
		CM	-1.38
	SPF	SPFp	1.24
		SPFm	0.77
	MED	PR	0.65
		MD	-0.48
		SMT	-1.25
	VENT	VM	0.13
		VAL	-0.09
		SPA	-0.2
		PoT	-0.59
		VPL	-1.29
		VPL	-1.36
	LAT	LP	0.02
		POL	-0.08
	MTN	Xi	-0.13
		RE	-0.43
	GENv	IntG	-0.24
		IGL	-0.98
		SubG	-1.01
		LGv	-1.03
	RT	RT	-0.29
	LH	LH	-0.79

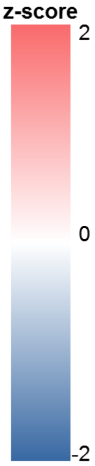

C

Region	Structure	Substructure	Z-score
Hypothalamus	MEZ	PH	0.38
		SUM	0.1
		PMd	-0.87
		PVHd	-0.93
		MM	-1.24
	LZ	ZI	1.91
		STN	-0.31
		PeF	-1.14
		PSTN	-1.18
		LHA	-1.19
		PST	-1.23

 © 2022 The Authors

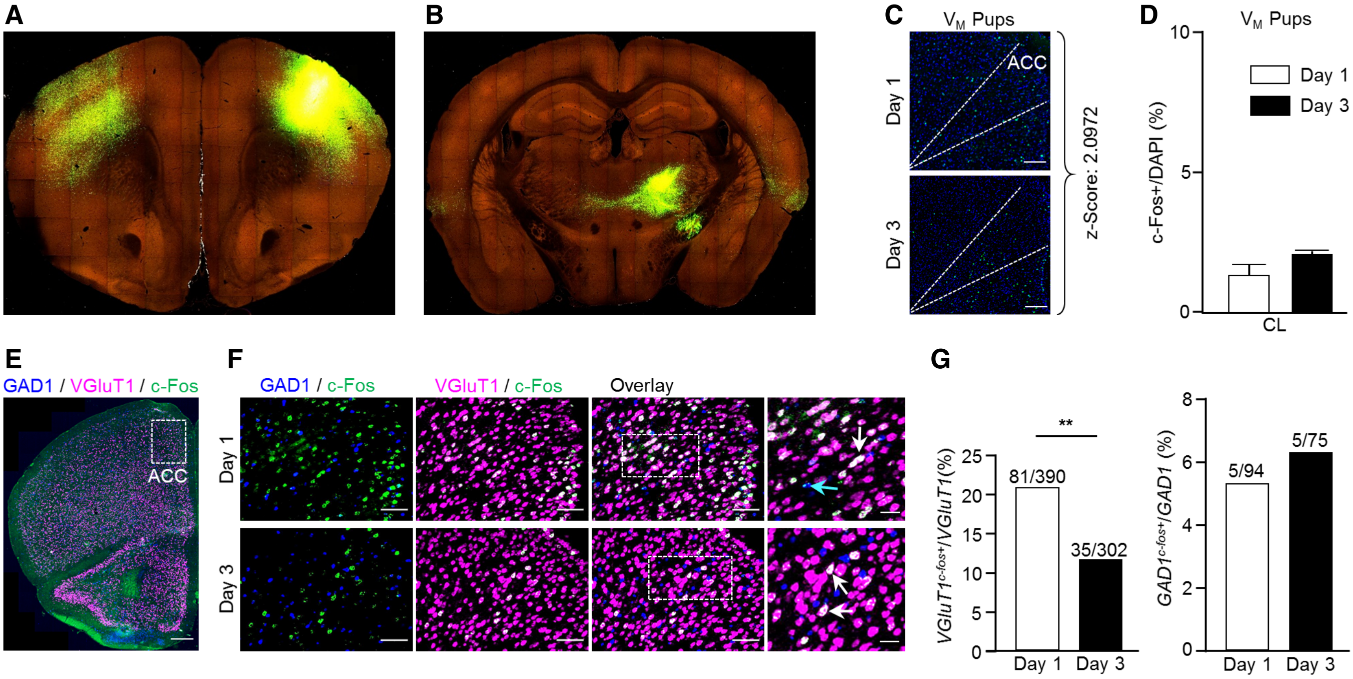

Figure EV3. Projections from the primary motor cortex to CM and PCN.

A, B Representative coronal images from the Allen Mouse Brain Connectivity Atlas (https://connectivity.brain-map.org/projection/experiment/100141780?imageId=102152345), following anterograde labeling with AAV-GFP in the primary motor cortex showing site of injection (A) and axonal distribution pattern in CM and PCN (B).

C Representative coronal sections of the mPFC immunostained for c-Fos with the anatomical borders of the ACC indicated by dashed lines, in virgin male mice on day 1 and 3 of behavioral testing. Scale bar 100 μm, 20× magnification. *Z*-score for the difference in the fraction of c-Fos-expressing cells, between day 1 and 3 of behavioral testing.

D Fraction of c-Fos^{+} cells in the CL central lateral nucleus in virgin male mice exposed to pups, on day 1 and 3 of behavioral testing (Day 1: $n = 4$, Day3: $n = 3$). Data were analyzed using Student's *t*-test and are expressed as mean ± s.e.m.

E, F A representative image of the PFC of a virgin female following initial pup exposure showing mRNA expression patterns of *slc17a7* (VGluT1), *gad1* (GAD1), and *c-fos* (c-Fos) together with high-magnification images of the ACC, following the 1st and 3rd days of pup-exposure. The scale bars 500 μm, 10× magnification (A), and 100 μm, 20× magnification (B).

G A comparison of the fraction of excitatory and inhibitory c-Fos^{+} cells, of the total VGluT1 and GAD1 cell population in the ACC, following the 1st and 3rd pup exposure days; data were analyzed using Fisher's exact test; $^{**}P < 0.01$.

Source data are available online for this figure.

© 2022 The Authors

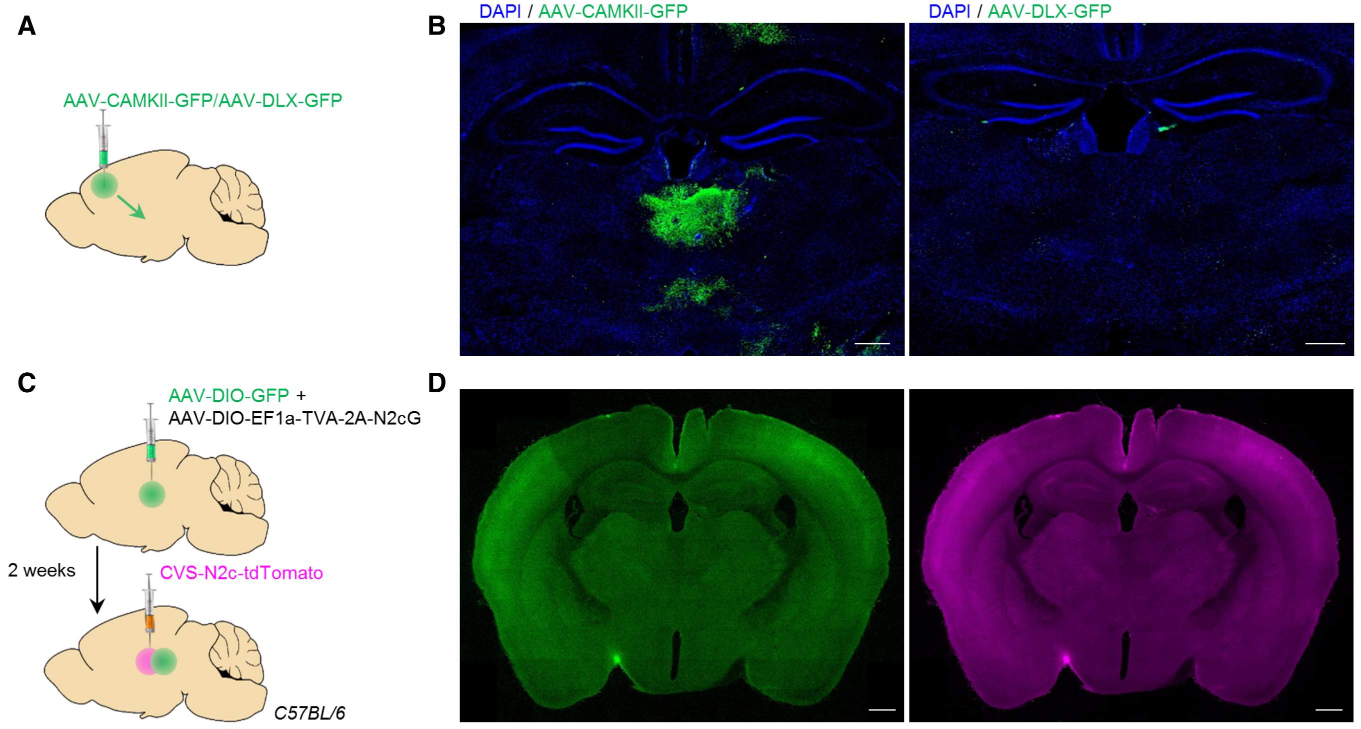

Figure EV4. Target specificity of viral vectors used for anterograde and retrograde labeling.

A Schematic representation of the anterograde tracing from ACC excitatory/inhibitory projections to thalamic structures.

B Representative coronal sections of the thalamic region, following the labeling schemes described in (A), for identification of axonal projections from excitatory (left)—but not inhibitory (right) ACC neurons. Scale bars 200 µm, 10× magnification.

C, D Schematic representation (C) and representative coronal sections (D) of the dual anterograde and retrograde tracing to and from the CL of C57BL/6 control mice. Scale bars 500 µm, 10× magnification.

 © 2022 The Authors

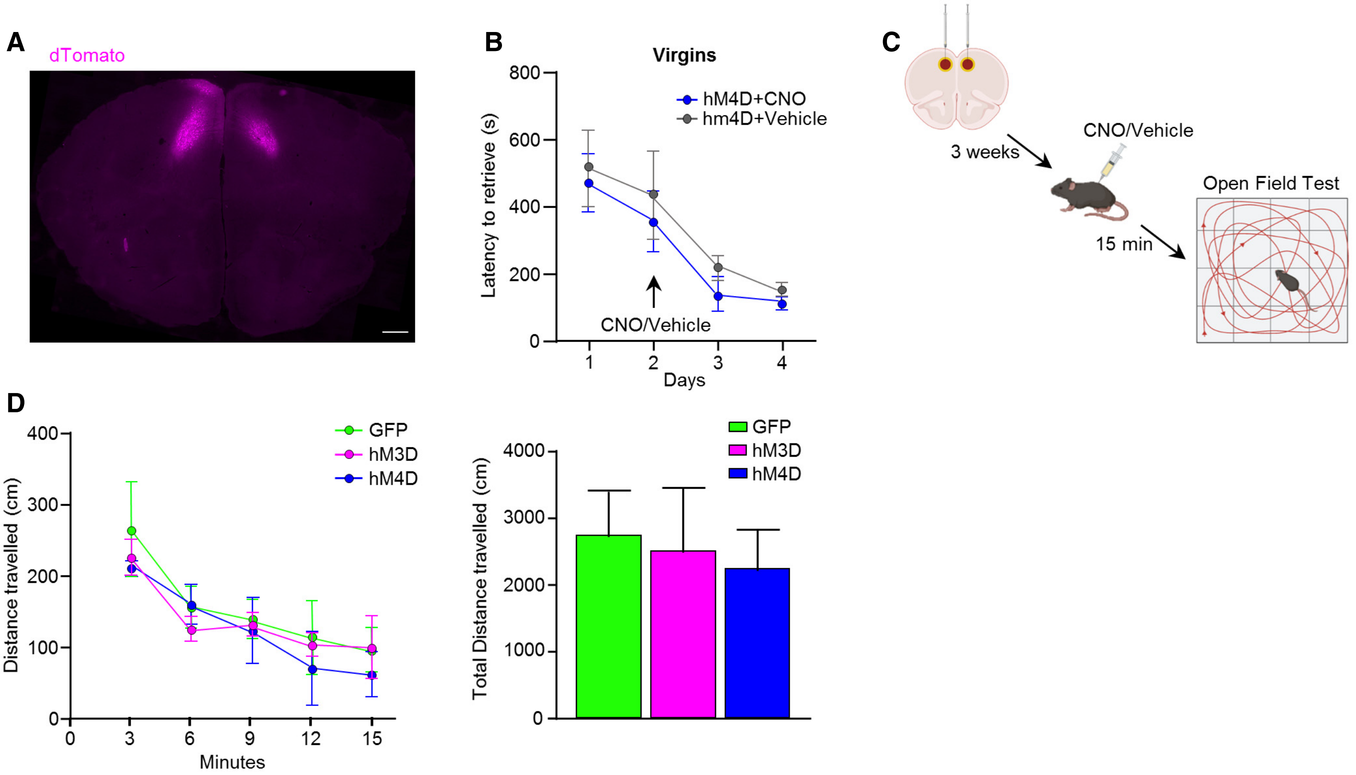

Figure EV5. Chemogenetic strategy.

A Representative coronal section demonstrating AAV-DIO-CAG-hM4D-2A-dTomato expression pattern in the ACC. Scale bar 500 μm, 10× magnification.

B Quantification of pup retrieval latencies of virgin females following CNO/vehicle administration on day 2 in mice injected with hM4D into the ACC. $n = 7$/group. Data were analyzed using two-way ANOVA with repeated measures and are expressed as mean ± s.e.m.

C Schematic representation of the chemogenetic strategy followed by the Open Field Test. Distance traveled in virgin females injected with hM3D/hM4D/GFP after CNO administration.

D Quantification of total distance traveled of virgin females in the Open Field Test following CNO/vehicle administration in mice injected with hM3D or hM4D into the ACC ($n = 5$/group). Data were analyzed using two-way ANOVA with repeated measures and are expressed as mean ± s.e.m.

Source data are available online for this figure.

© 2022 The Authors
