# Supplementary material for: An accessory prefrontal cortex–thalamus circuit sculpts maternal behavior in virgin female mice
Source: EMBO J. 2022 Nov 7;41(24):e111648. doi: 10.15252/embj.2022111648 (PMC9753463; doi:10.15252/embj.2022111648)
Supplement: Supplementary file 10 [file EMBJ-41-e111648-s004.pdf]

THE EMBO JOURNAL

An accessory prefrontal cortex-thalamus circuit sculpts maternal behavior in virgin female mice

Micaela Glat, Anna Gundacker, Laura Cuenca Rico, Barbara Czuczu, Yoav Ben Simon, Tibor Harkany, and Daniela Pollak
DOI: 10.15252/embj.2022111648

Corresponding author(s): Daniela Pollak (daniela.pollak@meduniwien.ac.at) , Tibor Harkany (tibor.harkany@meduniwien.ac.at), Daniela Pollak (daniela.pollak@meduniwien.ac.at)

Review Timeline:

Submission Date:	12th May 22
Editorial Decision:	20th Jun 22
Revision Received:	12th Sep 22
Editorial Decision:	5th Oct 22
Revision Received:	8th Oct 22
Accepted:	14th Oct 22

Editor: Karin Dumstrei

Transaction Report:

(Note: With the exception of the correction of typographical or spelling errors that could be a source of ambiguity, letters and reports are not edited. Depending on transfer agreements, referee reports obtained elsewhere may or may not be included in this compilation. Referee reports are anonymous unless the Referee chooses to sign their reports.)

1st Editorial Decision 20th Jun 2022

Dear Tibor,

Thank you for submitting your manuscript to The EMBO journal. Your study has now been seen by two referees and their comments are provided below.

As you can see the referees find the analysis interesting. However, they also raise several points that would be good to address in a revised version. I think it would be helpful to discuss the raised points further and I am available to do so via email or video.

When preparing your letter of response to the referees' comments, please bear in mind that this will form part of the Review Process File, and will therefore be available online to the community. For more details on our Transparent Editorial Process, please visit our website: https://www.embopress.org/page/journal/14602075/authorguide#transparentprocess

Thank you for the opportunity to consider your work for publication. I look forward to discussing your revisions further.

with best wishes

Karin

Karin Dumstrei, PhD
Senior Editor
The EMBO Journal

Guide For Authors: https://www.embopress.org/page/journal/14602075/authorguide

I have attached a guide with helpful tips on how to prepare the revised version.

We realise that it is difficult to revise to a specific deadline. In the interest of protecting the conceptual advance provided by the work, we recommend a revision within 3 months (18th Sep 2022).

As a matter of policy, competing manuscripts published during this period will not negatively impact on our assessment of the conceptual advance presented by your study. However, we request that you contact the editor as soon as possible upon publication of any related work, to discuss how to proceed.

If you require more time to complete the revisions let me know as as I can grant an extension.

Use the link below to submit your revision:

https://emboj.msubmit.net/cgi-bin/main.plex

Referee #1:

This paper tests a preconceived notion whether the accessory prefrontal cortex is involved in a behavior of virgin female mice that resembles caregiving to newborns. They provide evidence that it does and that it does and it does via the thalamus avoiding the medial preoptic area. This latter region is a known node that is involved in pregnancy-related development for maternal behavior.

The behavior that the authors aim to model in mice is a highly complex and ambiguous entity in humans, let alone in mice. The strategies they utilized are reasonable and logical and provide novel insights. Nevertheless, it is difficult to place the overall results in context of physiology, specifically that of the human condition. In addition, compared to humans, mice have a very limited development of the PFC.

Specific questions:

1) What iso the outcome of such induction of "maternal behavior" in future natural deliveries of the same mice?
2) Does it depend on the ovarian cycle how fast virgin females "acquire" this behavior?
3) Can males be induced to develop this maternal behavior?

Referee #2:

This study investigates maternal care in virgin females using pup retrieval behavior. The authors find that this behavior can be learned and that the ACC is a key mouse brain region activated during the behavior. By circuit tracing experiments, they find that the bidirectional and excitatory circuit between ACC and CL (thalamic centrolateral nucleus) is important, where Gal+ neurons in the CL stimulate excitatory neurons in the ACC. The authors also use chemogenetic methods to functionally validate the ACC-CL circuit.

This study employs careful and well-controlled approaches to identify and validate the key brain regions, cell types, and circuits associated with maternal caring in virgin females. Given that little is known about the mechanisms underlying the initial onset of maternal care behavior in virgin females, and that postpartum depression is a serious health issue, these findings are important and set a key stage for further studies.

Major comments:
1. The results from the chemogenetic experiments in Figure 4 do not seem to support the specific roles of the ACC-CL(Gal) pathway. The authors could try a pathway-specific modulation or optogenetic stimulation. At the very least, the authors need to give detailed interpretations of the current results with regard to cell-type specificity.

2. It is unclear why the authors did not try to modulate CL(Gal) neurons and see whether ACC neurons show altered activities and the mice show altered behavior. Such modulations would strongly support the reciprocal and functional connectivity between ACC and CL.

Minor comments:
1. In Figure 1, the authors show the extent of parental behavior and ignorance (Figure 1c,d) as percentages. The authors should clarify, in the methods section, the criteria of "ignoring" behavior, as parental and ignoring behaviors do not add up to 100%.

2. Figures 4b-d are not easy to read. I wonder whether the authors could show the results from individual days by ANOVA comparisons to clearly visualize the differences.

3. It will help readers if the authors could add detailed labels of the brain regions shown in Figures 2 and 3 panels.

4. Would the ACC-CL circuit also contribute to the acquisition of parenting behavior in male mice?

Response to the Referees' comments on the manuscript:
"An accessory prefrontal cortex- thalamus circuit sculpts maternal behavior in virgin female mice" (EMBOJ-2022-111648)

Referee #1:

This paper tests a preconceived notion whether the accessory prefrontal cortex is involved in a behavior of virgin female mice that resembles caregiving to newborns. They provide evidence that it does and it does via the thalamus avoiding the medial preoptic area. This latter region is a known node that is involved in pregnancy-related development for maternal behavior. The behavior that the authors aim to model in mice is a highly complex and ambiguous entity in humans, let alone in mice. The strategies they utilized are reasonable and logical and provide novel insights. Nevertheless, it is difficult to place the overall results in context of physiology, specifically that of the human condition. In addition, compared to humans, mice have a very limited development of the PFC.

We thank the Referee for the overall very positive evaluation of our manuscript, the experimental approach and the results. We are grateful to the Referee for pointing out that the need to better conceptualize our data and the overall message within the context of physiology and to specify its human relevance, if any. We have integrated these aspects into the revised manuscript in the discussion section (*blue highlights*). In the respective paragraph on p.12, we propose the identified ACC-CL loop to operate as an alternative route to activate the maternal care circuit, specifically the PAG for the final motor execution of this behavioural repertoire. As such, we suggest this *additional circuit element to act as an enhancer to compensate for the absence of hormonal priming of the mPOA in virgin females* and to enable the experience-dependent acquisition of pup retrieval behavior. In the broader context of physiology and pathobiology, our observations suggest that *complementary (or even redundant) mechanisms exist to ensure engagement in offspring care via learnt strategies*. From a translational perspective, our findings suggest that in conditions when mothers cannot readily take on offspring care because of a constellation of exogenous and/or endogenous influences, behavioural performance can be enhanced by recruiting non-pregnancy/non-parturition-related learning strategies.

Specific questions:

1) What is the outcome of such induction of "maternal behavior" in future natural deliveries of the same mice?

The point raised by the Referee touches on an interesting question. Our experimental design was based upon the pup retrieval test as robust and reliable readout for the evaluation of maternal behaviour. In the current settings, mothers retrieve the pups very fast. It would be unlikely that we could detect a further "improvement" in their performance, which has previously been used for the

induction of maternal behaviour as virgins (floor effect). Therefore, and considering that we suggest this being a learnt behavior, *our hypothesis is that once learning is consolidated, the animals will be able to retrieve their repertoire without recruiting this neurocircuit again*.

2) Does it depend on the ovarian cycle how fast virgin females "acquire" this behavior?

The Referee indeed put forward a highly relevant question, which we have experimentally addressed during the revision process. Indeed, it is perceivable that the distinct hormonal levels/profiles associated with individual stages of the ovarian cycle could bias how fast virgin females acquire parental behavior. Therefore, *we grouped females according to their cycle stage ("follicular stage": pre-estrus/ estrus; "secretory stage" metestrus/ diestrus) at their first pup exposure.* We found no difference between the groups, suggesting that the *hormonal phase indeed did not bias the performance of virgin females*. These data are shown in **Extended View Fig. 1a** (revised numbering).

3) Can males be induced to develop this maternal behavior?

We thank the referee for another stimulating question, which is echoed by Referee #2. Indeed, there is evidence in the literature that male mice can develop parental behaviour, although their response to pups is highly dependent on their endogenous state (*see for review*: PMID 26122293). While fathers will exhibit parental care behaviour (PMID 23299896), including pup retrieval, it is well documented that virgin male mice display aggressive behaviour towards pups, most typically resulting in them attacking and finally killing the pups. This occurs because the evolutionary goal is to remove the current litter. This is achieved by stopping the female to lactate, thus accelerating the time to her next ovulation and enhancing the probability of a successful mating (PMID 7058349). Against the background of animal welfare and ethical considerations, we have decided to address your question in a setting not involving potential physical harm to the pups with their retrieval being a major outcome parameter. Instead, and also responding to a similar request by Referee #2, we have examined whether repeated contact with pups over three consecutive days (with the pups being protected from the physical attacks by the males in a wire mesh enclosure, which however allowed the male to use all classical sensory cues) would lead to an engagement of the ACC-CL circuit, comparable to the one of virgin females. We *have found that the distinctive change in ACC c-Fos labelling across days in virgin females does not occur in males.* Thus, we suggest sex-specific activation patterns. Nevertheless, we think these data could be over-interpreted if presented in the core manuscript. Therefore, we placed them in **Extended View Fig. 4** (revised numbering).

Referee #2:

This study investigates maternal care in virgin females using pup retrieval behavior. The authors find that this behavior can be learned and that the ACC is a key mouse brain region activated during the behavior. By circuit tracing experiments, they find that the bidirectional and excitatory circuit between ACC and CL (thalamic centrolateral nucleus) is important, where Gal^{+} neurons in the CL stimulate excitatory neurons in the ACC. The authors also use chemogenetic methods to functionally validate the ACC-CL circuit.

This study employs careful and well-controlled approaches to identify and validate the key brain regions, cell types, and circuits associated with maternal caring in virgin females. Given that little is known about the mechanisms underlying the initial onset of maternal care behavior in virgin females, and that postpartum depression is a serious health issue, these findings are important and set a key stage for further studies.

We sincerely appreciate the favourable feedback on our manuscript and its relevance. We have considered all points raised by the Referee as detailed below.

Major comments:

1. The results from the chemogenetic experiments in Figure 4 do not seem to support the specific roles of the ACC-CL(Gal) pathway. The authors could try a pathway-specific modulation or optogenetic stimulation. At the very least, the authors need to give detailed interpretations of the current results with regard to cell-type specificity.

Indeed, the aspect of cell-type specificity is highly pertinent to the overall findings presented in our manuscript. We thank the Referee for motivating us to further explore this question. Therefore, we assessed the consequences of the specific chemogenetic activation of CL^{Gal+} neurons on pup retrieval behavior in virgins, using *Gal-Cre mice* (that are widely used by the community in studies on hypothalamic organization and function). Remarkably, *we found that chemogenetic activation of CL^{Gal+} neurons in virgin females significantly shortened the latency to retrieve the pups, as compared to controls*. These results corroborate those we have presented in the original manuscript (non-cell-type-specific manipulations). Data are now part of **revised Figure 6,** and support the specific engagement of the ACC-CL(Gal) pathway for the display of maternal behaviour in virgin females.

2. It is unclear why the authors did not try to modulate CL(Gal) neurons and see whether ACC neurons show altered activities and the mice show altered behavior. Such modulations would strongly support the reciprocal and functional connectivity between ACC and CL.

As described above, the chemogenetic *modulation of CL^{Gal+} neurons alone was indeed sufficient to bias the behaviours of virgin females*.

Minor comments:

1. In Figure 1, the authors show the extent of parental behavior and ignorance (Figure 1c,d) as percentages. The authors should clarify, in the methods section, the criteria of "ignoring" behavior, as parental and ignoring behaviors do not add up to 100%

We thank the Referee for drawing our attention to this point. We have clarified the terminology and are now using the terms '*pup-directed*' (instead of 'parental') and '*non-pup directed*' (instead of 'ignoring') behavior in the respective description of the results on p.4 and in the corresponding methods. There, we also defined that pup-directed behaviors include crouching, licking and grooming, and covering over the pups. In contrast, non-pup directed behaviors comprise self-grooming, eating and sleeping. Other elements related to parental care, but not involving physical interaction with the pups (eg. nest building), were not considered explaining why pup-directed and non-pup-directed behaviors do not add up to 100%.

2. Figures 4b-d are not easy to read. I wonder whether the authors could show the results from individual days by ANOVA comparisons to clearly visualize the differences.

Following the Referee's suggestion, we have changed the graphical representations in Figures 4b-4d to better display the differences between the individual days.

3. It will help readers if the authors could add detailed labels of the brain regions shown in Figures 2 and 3 panels.

As proposed by the Referee, we have added detailed labels of the brain regions shown in Figures 2 and 3.

4. Would the ACC-CL circuit also contribute to the acquisition of parenting behavior in male mice?

The Referee raises an interesting question, which relates to a comment brought up by Referee 1 (#3). Indeed, there is evidence in the literature that male mice can develop parental behaviour, although their response to pups is highly dependent on their endogenous state (see for review: PMID 26122293). While fathers will exhibit parental care behaviour (PMID 23299896), including

pup retrieval, it is well documented that virgin male mice display aggressive behaviour towards pups, most typically resulting in them attacking and finally killing the pups. The evolutionary goal is to remove the current litter and therefore stop lactation of the female. This is expected to accelerate the time to her next ovulation, enhancing the probability of a successful mating thereafter (PMID 7058349). Against the background of animal welfare and ethical considerations, we have decided to address your question in a setting not involving potential physical harm to the pups with their retrieval being a major outcome parameter. Instead, we have examined whether repeated contact with pups over three consecutive days (with the pups being protected from the physical attacks by the males in a wire mesh enclosure, which however allowed the male to use all classical sensory cues) would lead to an engagement of the ACC-CL circuit, comparable to the one of virgin females. We *have found that the distinctive change in ACC c-Fos labelling across days in virgin females does not occur in males.* Thus, we suggest sex-specific activation patterns. Nevertheless, we think these data could be over-interpreted if presented in the core manuscript. Therefore, we placed them in **Extended View Fig. 4** (revised numbering).

Overall, we thank the expert Referees their comments. We hope our answers will be accepted, particularly given the experimental follow-up we have carried out during the revision process.

Yours sincerely,

Daniela Pollak and Tibor Harkany

1st Revision - Editorial Decision **5th Oct 2022**

Dear Tibor,

Thank you for submitting your revised manuscript to The EMBO Journal. Your study has now been re-reviewed by referee #2 who appreciates the introduced changes. I am therefore very pleased to let you know that we will accept the manuscript for publication here. Before sending you the formal accept letter there are a few editorial points that need to be resolved.

- we are missing 3-5 keywords

- COI needs to be renamed to Disclosure and Competing Interests Statement

- Please remove the Authors Contributions from the manuscript. The 'Author Contributions' section is replaced by the CRediT contributor roles taxonomy to specify the contributions of each author in the journal submission system. Please use the free text box in the 'author information' section of the manuscript submisssion system to provide more detailed descriptions (e.g., 'X provided intracellular Ca++ measurements in fig Y')

- Make sure that the author checklist is completely filled out.

- Please also check panel callouts for Figure 7. Supplementary Data Table 1 is called out, but there is no Supplementary Data Table 1.

- The source data needs to be sorted as one folder per figure and ZIPed together. If you have source data for EV figures, please ZIP them together in a separate folder. See also https://www.embopress.org/pb-assets/embo-site/Guide%20for%20SourceData%20Submission-1656066810500.pdf

- We include a synopsis of the paper (see http://emboj.embopress.org/). Please provide me with a general summary statement and 3-5 bullet points that capture the key findings of the paper.

- We also need a summary figure for the synopsis. The size should be 550 wide by [200-400] high (pixels).

- Figure 2-D3 zoom box. Please check that the "zoom" box matches the highlighted square

- Figure 4B - please check the labeling of the "zoom". I think it should be 1,2,3. Please also double check that the "zoom" box matches the highlighted box.

- Figure 6B - the highlighted box has been drawn to wide. Please redraw to reflect the zoom box.

- There are some black boxes in Figure EV5 B -Day 1 panels.

- you can only have 5 EV figures. I think EV3, EV4 & EV5 can be combined into one EV figure

- The legend for Table EV1 should also be added to the ms file

- The author email bounced for Laura Cuenca Rico - laura.cuenca-rico@meduniwien.ac.at - please double check.

- Also, can you make sure to upload good resolution figures

- Our publisher has also done their pre-publication check on your manuscript. When you log into the manuscript submission system you will see the file "Data Edited Manuscript file". Please look at the word file and the comments regarding the figure legends and respond to the issues.

- Please also include a point-by-point response to the editorial points when you resubmit the revised version.

That should be all - let me know if you have any further questions. And congratulations on a nice study!

With best wishes

Karin

Karin Dumstrei, PhD
Senior Editor
The EMBO Journal

Use the link below to submit your revision:

https://emboj.msubmit.net/cgi-bin/main.plex

Referee #2:

The authors have addressed all of my review comments. The results from the chemogenetic modulation of CLGal+ neurons are convincing, and also the results from male mice are interesting. I do not have any additional comments.

Dear Karin, please find below our replies to you editorial comments.

Thank you so much for your support,

Best regards,

Daniela and Tibor

Response to the Editorial comments on the manuscript:

"An accessory prefrontal cortex- thalamus circuit sculpts maternal behavior in virgin female mice" (EMBOJ-2022-111648R1)

1. We are missing 3-5 keywords.
The key words have been added after the abstract.

2. COI needs to be renamed to Disclosure and Competing Interests Statement.
COI has been renamed accordingly.

3. Please remove the Authors Contributions from the manuscript. The 'Author Contributions' section is replaced by the CRediT contributor roles taxonomy to specify the contributions of each author in the journal submission system. Please use the free text box in the 'author information' section of the manuscript submisssion system to provide more detailed descriptions.
The Authors contributions have been removed from the manuscript and the respective section in the journal submission system amended where necessary.

4. Make sure that the author checklist is completely filled out.
The author checklist has been checked and completed where required.

5. Please also check panel callouts for Figure 7. Supplementary Data Table 1 is called out, but there is no Supplementary Data Table 1.
The panel callouts for Figure 7 have been checked and the call out for Supplementary Data Table 1 removed from the text.

6. The source data needs to be sorted as one folder per figure and ZIPed together. If you have source data for EV figures, please ZIP them together in a separate folder.
The source data have been sorted and ZIPed as indicated.

7. We include a synopsis of the paper (see http://emboj.embopress.org/). Please provide me with a general summary statement and 3-5 bullet points that capture the key findings of the paper.
A general summary statement and corresponding bullets points are now included in the submission.

8. We also need a summary figure for the synopsis. The size should be 550 wide by [200-400] high (pixels).
A summary figure with the size guidelines provided has been uploaded.

9. Figure 2-D3 zoom box. Please check that the "zoom" box matches the highlighted square.
The zoom box in Figure 2-D3 has been checked and corrected.

10. Figure 4B - please check the labeling of the "zoom". I think it should be 1,2,3. Please also double check that the "zoom" box matches the highlighted box.
The labelling of the zoom boxes and their matching with the highlighted box has been checked and corrected for Figure 4B.

11. Figure 6B - the highlighted box has been drawn to wide. Please redraw to reflect the zoom box.
The highlighted box in Figure 6B has been redrawn to reflect the zoom box.

12. There are some black boxes in Figure EV5 B -Day 1 panels.
The black boxes in Figure EV5B – Day1 panels have been removed.

13. You can only have 5 EV figures. I think EV3, EV4 & EV5 can be combined into one EV figure.
The EV figures have been reduced to five, as EV3/ EV4 and EV5 have been combined into one EV figure as suggested.

14. The legend for Table EV1 should also be added to the ms file.
The legend for Table EV1 has been added to the ms file.

15. The author email bounced for Laura Cuenca Rico.
The author email for Laura Cuenca Rico has been checked and corrected.

16. Also, can you make sure to upload good resolution figures.
The resolution of the figures has been improved and good resolution figures have been uploaded.

17. Our publisher has also done their pre-publication check on your manuscript. When you log into the manuscript submission system you will see the file "Data Edited Manuscript file". Please look at the word file and the comments regarding the figure legends and respond to the issues.
We have checked the Data Edited Manuscript word file and responded to all issues raised.

Dear Daniela and Tibor,

Thank you for submitting your revised manuscript. I have now had a chance to look at the revised version and all looks good!

I am therefore very pleased to accept the manuscript for publication here.

Congratulations on a nice study!

With best wishes

Karin

Karin Dumstrei, PhD
Senior Editor
The EMBO Journal

Please note that it is EMBO Journal policy for the transcript of the editorial process (containing referee reports and your response letter) to be published as an online supplement to each paper. If you do NOT want this, you will need to inform the Editorial Office via email immediately. More information is available here: https://www.embopress.org/page/journal/14602075/authorguide#transparentprocess

Your manuscript will be processed for publication in the journal by EMBO Press. Manuscripts in the PDF and electronic editions of The EMBO Journal will be copy edited, and you will be provided with page proofs prior to publication. Please note that supplementary information is not included in the proofs.

You will be contacted by Wiley Author Services to complete licensing and payment information. The required 'Page Charges Authorization Form' is available here: https://www.embopress.org/pb-assets/embo-site/tej_apc.pdf - please download and complete the form and return to embopressproduction@wiley.com

EMBO Press participates in many Publish and Read agreements that allow authors to publish Open Access with reduced/no publication charges. Check your eligibility: https://authorservices.wiley.com/author-resources/Journal-Authors/open-access/affiliation-policies-payments/index.html

Should you be planning a Press Release on your article, please get in contact with embojournal@wiley.com as early as possible, in order to coordinate publication and release dates.

If you have any questions, please do not hesitate to call or email the Editorial Office. Thank you for your contribution to The EMBO Journal.

** Click here to be directed to your login page: https://emboj.msubmit.net

EMBO Press Author Checklist

Corresponding Author Name: Pollak Daniela D.	
Journal Submitted to: The EMBO journal	
Manuscript Number: EMBOJ-2022-111648	

USEFUL LINKS FOR COMPLETING THIS FORM
The EMBO Journal - Author Guidelines
EMBO Reports - Author Guidelines
Molecular Systems Biology - Author Guidelines
EMBO Molecular Medicine - Author Guidelines

Reporting Checklist for Life Science Articles (updated January
This checklist is adapted from Materials Design Analysis Reporting (MDAR) Checklist for Authors. MDAR establishes a minimum set of requirements in transparent reporting in the life sciences (see Statement of Task: 10.31222/osf.io/9sm4x). Please follow the journal's guidelines in preparing your
Please note that a copy of this checklist will be published alongside your article.

Abridged guidelines for figures
1. Data
The data shown in figures should satisfy the following conditions:
- the data were obtained and processed according to the field's best practice and are presented to reflect the results of the experiments in an accurate and unbiased manner.
- ideally, figure panels should include only measurements that are directly comparable to each other and obtained with the same assay.
- plots include clearly labeled error bars for independent experiments and sample sizes. Unless justified, error bars should not be shown for technical
- if n<5, the individual data points from each experiment should be plotted. Any statistical test employed should be justified.
- Source Data should be included to report the data underlying figures according to the guidelines set out in the authorship guidelines on Data

2. Captions
Each figure caption should contain the following information, for each panel where they are relevant:
- a specification of the experimental system investigated (eg cell line, species name).
- the assay(s) and method(s) used to carry out the reported observations and measurements.
- an explicit mention of the biological and chemical entity(ies) that are being measured.
- an explicit mention of the biological and chemical entity(ies) that are altered/varied/perturbed in a controlled manner.
- the exact sample size (n) for each experimental group/condition, given as a number, not a range;
- a description of the sample collection allowing the reader to understand whether the samples represent technical or biological replicates (including how many animals, litters, cultures, etc.).
- a statement of how many times the experiment shown was independently replicated in the laboratory.
- definitions of statistical methods and measures:
  - common tests, such as t-test (please specify whether paired vs. unpaired), simple χ2 tests, Wilcoxon and Mann-Whitney tests, can be unambiguously identified by name only, but more complex techniques should be described in the methods section;
  - are tests one-sided or two-sided?
  - are there adjustments for multiple comparisons?
  - exact statistical test results, e.g., P values = x but not P values < x;
  - definition of 'center values' as median or average;
  - definition of error bars as s.d. or s.e.m.

Please complete ALL of the questions below.
Select "Not Applicable" only when the requested information is not relevant for your study.

Materials

Newly Created Materials	Information included in the manuscript?	In which section is the information available? (Reagents and Tools Table, Materials and Methods, Figures, Data Availability Section)
New materials and reagents need to be available; do any restrictions apply?	Not Applicable	

Antibodies	Information included in the manuscript?	In which section is the information available? (Reagents and Tools Table, Materials and Methods, Figures, Data Availability Section)
For **antibodies** provide the following information:<br>- Commercial antibodies: RRID (if possible) or supplier name, catalogue number and or/clone number<br>- Non-commercial: RRID or citation	Not Applicable	

DNA and RNA sequences	Information included in the manuscript?	In which section is the information available? (Reagents and Tools Table, Materials and Methods, Figures, Data Availability Section)
**Short novel DNA or RNA including primers, probes**: provide the sequences.	Not Applicable	

Cell materials	Information included in the manuscript?	In which section is the information available? (Reagents and Tools Table, Materials and Methods, Figures, Data Availability Section)
**Cell lines:** Provide species information, strain. Provide accession number in repository **OR** supplier name, catalog number, clone number, and/**OR** RRID.	Not Applicable	
**Primary cultures:** Provide species, strain, sex of origin, genetic modification status.	Not Applicable	
Report if the cell lines were recently **authenticated** (e.g., by STR profiling) and tested for mycoplasma contamination.	Not Applicable	

Experimental animals	Information included in the manuscript?	In which section is the information available? (Reagents and Tools Table, Materials and Methods, Figures, Data Availability Section)
**Laboratory animals or Model organisms:** Provide species, strain, sex, age, genetic modification status. Provide accession number in repository **OR** supplier name, catalog number, clone number, **OR** RRID.	Yes	Materials and Methods
**Animal observed in or captured from the field:** Provide species, sex, and age where possible.	Not Applicable	
Please detail **housing and husbandry conditions**.	Yes	Materials and Methods

Plants and microbes	Information included in the manuscript?	In which section is the information available? (Reagents and Tools Table, Materials and Methods, Figures, Data Availability Section)
**Plants:** provide species and strain, ecotype and cultivar where relevant, unique accession number if available, and source (including location for collected wild specimens).	Not Applicable	
**Microbes:** provide species and strain, unique accession number if available, and source.	Not Applicable	

Human research participants	Information included in the manuscript?	In which section is the information available? (Reagents and Tools Table, Materials and Methods, Figures, Data Availability Section)
If collected and within the bounds of privacy constraints report on age, sex and gender or ethnicity for all study participants.	Not Applicable	

Core facilities	Information included in the manuscript?	In which section is the information available? (Reagents and Tools Table, Materials and Methods, Figures, Data Availability Section)
If your work benefited from core facilities, was their service mentioned in the acknowledgments section?	Not Applicable	

Design

Study protocol	Information included in the manuscript?	In which section is the information available? (Reagents and Tools Table, Materials and Methods, Figures, Data Availability Section)
If study protocol has been **pre-registered, provide DOI in the manuscript**. For clinical trials, provide the trial registration number **OR** cite DOI.	Not Applicable	
Report the **clinical trial registration number** (at ClinicalTrials.gov or equivalent), where applicable.	Not Applicable	

Laboratory protocol	Information included in the manuscript?	In which section is the information available? (Reagents and Tools Table, Materials and Methods, Figures, Data Availability Section)
Provide DOI OR other citation details if **external detailed step-by-step protocols** are available.	Not Applicable	

Experimental study design and statistics	Information included in the manuscript?	In which section is the information available? (Reagents and Tools Table, Materials and Methods, Figures, Data Availability Section)
Include a statement about **sample size** estimate even if no statistical methods were used.	Yes	Materials and Methods
Were any steps taken to minimize the effects of subjective bias when allocating animals/samples to treatment (e.g. **randomization procedure**)? If yes, have they been described?	Not Applicable	
Include a statement about **blinding** even if no blinding was done.	Yes	Materials and Methods
Describe **inclusion/exclusion criteria** if samples or animals were excluded from the analysis. Were the criteria pre-established?<br><br>If sample or data points were omitted from analysis, report if this was due to attrition or intentional exclusion and provide justification.	Not Applicable	
For every figure, are **statistical tests** justified as appropriate? Do the data meet the assumptions of the tests (e.g., normal distribution)? Describe any methods used to assess it. Is there an estimate of variation within each group of data? Is the variance similar between the groups that are being statistically compared?	Yes	Materials and Methods

Sample definition and in-laboratory replication	Information included in the manuscript?	In which section is the information available? (Reagents and Tools Table, Materials and Methods, Figures, Data Availability Section)
In the figure legends: state number of times the experiment was **replicated** in laboratory.	Yes	
In the figure legends: define whether data describe **technical or biological replicates**.	Yes	

Ethics

Ethics	Information included in the manuscript?	In which section is the information available? (Reagents and Tools Table, Materials and Methods, Figures, Data Availability Section)
Studies involving **human participants**: State details of **authority granting ethics approval** (IRB or equivalent committee(s), provide reference number for approval.	Not Applicable	
Studies involving **human participants**: Include a statement confirming that **informed consent** was obtained from all subjects and that the experiments conformed to the principles set out in the WMA Declaration of Helsinki and the Department of Health and Human Services Belmont Report.	Not Applicable	
Studies involving **human participants:** For publication of **patient photos**, include a statement confirming that consent to publish was obtained.	Not Applicable	
Studies involving experimental **animals**: State details of **authority granting ethics approva**l (IRB or equivalent committee(s), provide reference number for approval. Include a statement of compliance with ethical regulations.	Yes	Materials and Methods
Studies involving **specimen and field samples:** State if relevant **permits** obtained, provide details of authority approving study; if none were required, explain why.	Not Applicable	

Dual Use Research of Concern (DURC)	Information included in the manuscript?	In which section is the information available? (Reagents and Tools Table, Materials and Methods, Figures, Data Availability Section)
Could your study fall under dual use research restrictions? Please check biosecurity documents and list of **select agents and toxins** (CDC): https://www.selectagents.gov/sat/list.htm	Not Applicable	
If you used a select agent, is the security level of the lab appropriate and reported in the manuscript?	Not Applicable	
If a study is subject to dual use research of concern regulations, is the name of the **authority granting approval and reference number** for the regulatory approval provided in the manuscript?	Not Applicable	

Reporting

The MDAR framework recommends adoption of discipline-specific guidelines, established and endorsed through community initiatives. Journals have their own policy about requiring specific guidelines and recommendations to complement MDAR.

Adherence to community standards	Information included in the manuscript?	In which section is the information available? (Reagents and Tools Table, Materials and Methods, Figures, Data Availability Section)
State if relevant guidelines or checklists (e.g., **ICMJE, MIBBI, ARRIVE, PRISMA**) have been followed or provided.	Not Applicable	
For **tumor marker prognostic studies,** we recommend that you follow the **REMARK** reporting guidelines (see link list at top right). See author guidelines, under 'Reporting Guidelines'. Please confirm you have followed these guidelines.	Not Applicable	
For **phase II and III randomized controlled trials**, please refer to the **CONSORT** flow diagram (see link list at top right) and submit the CONSORT checklist (see link list at top right) with your submission. See author guidelines, under 'Reporting Guidelines'. Please confirm you have submitted this list.	Not Applicable	

Data Availability

Data availability	Information included in the manuscript?	In which section is the information available? (Reagents and Tools Table, Materials and Methods, Figures, Data Availability Section)
Have **primary datasets** been deposited according to the journal's guidelines (see 'Data Deposition' section) and the respective accession numbers provided in the Data Availability Section?	Not Applicable	
Were **human clinical and genomic datasets** deposited in a public access-controlled repository in accordance to ethical obligations to the patients and to the applicable consent agreement?	Not Applicable	
Are **computational models** that are central and integral to a study available without restrictions in a machine-readable form? Were the relevant accession numbers or links provided?	Not Applicable	
If publicly available data were reused, provide the respective **data citations in the reference list**.	Not Applicable	
